# “Complimenting the Complement”: Mechanistic Insights and Opportunities for Therapeutics in Hepatocellular Carcinoma

**DOI:** 10.3389/fonc.2020.627701

**Published:** 2021-02-24

**Authors:** Astha Malik, Unmesha Thanekar, Surya Amarachintha, Reena Mourya, Shreya Nalluri, Alexander Bondoc, Pranavkumar Shivakumar

**Affiliations:** ^1^ Division of Gastroenterology, Hepatology and Nutrition, Cincinnati Children’s Hospital Medical Center, Cincinnati, OH, United States; ^2^ Department of Pediatrics, University of Cincinnati College of Medicine, Cincinnati, OH, United States; ^3^ Division of Pediatric General and Thoracic Surgery, Cincinnati Children’s Hospital Medical Center, Cincinnati, OH, United States

**Keywords:** hepatocellular carcinoma, HCC and COVID-19, complement activation, complement proteins, prognostic markers, inflammatory factors, complement-based therapeutics, immunotherapy

## Abstract

Hepatocellular carcinoma (HCC) is the most common primary malignancy of the liver and a leading cause of death in the US and worldwide. HCC remains a global health problem and is highly aggressive with unfavorable prognosis. Even with surgical interventions and newer medical treatment regimens, patients with HCC have poor survival rates. These limited therapeutic strategies and mechanistic understandings of HCC immunopathogenesis urgently warrant non-palliative treatment measures. Irrespective of the multitude etiologies, the liver microenvironment in HCC is intricately associated with chronic necroinflammation, progressive fibrosis, and cirrhosis as precedent events along with dysregulated innate and adaptive immune responses. Central to these immunological networks is the complement cascade (CC), a fundamental defense system inherent to the liver which tightly regulates humoral and cellular responses to noxious stimuli. Importantly, the liver is the primary source for biosynthesis of >80% of complement components and expresses a variety of complement receptors. Recent studies implicate the complement system in liver inflammation, abnormal regenerative responses, fibrosis, carcinogenesis, and development of HCC. Although complement activation differentially promotes immunosuppressive, stimulant, and angiogenic microenvironments conducive to HCC development, it remains under-investigated. Here, we review derangement of specific complement proteins in HCC in the context of altered complement regulatory factors, immune-activating components, and their implications in disease pathogenesis. We also summarize how complement molecules regulate cancer stem cells (CSCs), interact with complement-coagulation cascades, and provide therapeutic opportunities for targeted intervention in HCC.

## HCC: Incidence, Etiology, and Treatment

Hepatocellular Carcinoma (HCC) is the major form of primary malignancy of the liver, derived mostly from hepatocytes in more than 80% of the cases. HCC ranks as the fifth most common cancer in men and the seventh in women, representing a third of all cancer-related deaths (1) and centralizing mostly in developing countries. Globally, the incidence of HCC continues to rise, with rates increasing from 2.7/100,000 in 1997 to 8.8/100,000 in 2016 in men and from 0.8/100,000 to 2.2/100,000 in women. HCC is associated with unfavorable trends in North America, Northern and Central Europe, and Latin America. Development of HCC with enhanced tumor burden is highly prevalent in patients with liver cirrhosis as the single-most important etiology (2, 3). While HCC uniformly results in high mortality, the etiology and epidemiology differ widely in their geographical distributions. In western countries, including USA, and in Japan, chronic hepatitis C virus (HCV) infection is the primary risk factor (4, 5) and hepatitis B virus (HBV) infection is more prevalent in Southeast Asia, China, and sub-Saharan Africa (6). Since liver cirrhosis underpins the fundamental cause of HCC, patients with chronic liver diseases and a predisposition to cirrhosis are at substantial risk (7). However, contributions from nonalcoholic steatohepatitis (NASH), diabetes mellitus, obesity, and autoimmune and cholestatic diseases as predisposing factors in the onset of HCC are relatively minor (8). In contrast, an alarming rise in HCV, alcohol-related, and post-NASH HCC has been found in the United States, Canada, areas of Europe, Australia, and New Zealand (9, 10).

As an aggressive disease typified frequently by late diagnosis, the prognosis for HCC remains very poor (7), with median survival following diagnosis ranging from 6 to 20 months (11) and a 5-year relative survival rate of 18.4%. Cirrhosis and portal vein occlusion define the length of survival and severely limit therapeutic options, resulting in liver failure, tumor progression, and death. The existence of underlying advanced chronic liver disease, tumor stage, and portal hypertension in most of patients with HCC dictates and complicates the adoption of treatment strategies and prognosis. Treatment options including medical and transplantation for large non-resectable HCC patients, unfortunately, share high tumor recurrence rates due to persistent cirrhosis that confers a preneoplastic environment (12). The only curative treatment strategies involve orthotopic liver transplantation (OLT) and surgical liver resections (LR). OLT, however, is limited by organ shortage, resulting in increased utilization of extended-criteria donor (ECD) allografts (13). Other surgical interventions include but are not limited to the locoregional tumor ablation therapies including TACE (14), trans-arterial radioembolization with Yttrium-90 (Y-90) (15), stereotactic body radiotherapy (SBRT) (16), percutaneous ethanol injection (PEI) (17), high-intensity focused ultrasound (HIFU) (18), irreversible electroporation (IRE) (19), and radiofrequency-, microwave-, and cryo- ablations (19, 20). While surgical therapy remains the mainstay of treating HCC, systemic treatments targeting the molecular signaling pathways are frequently implemented for patients with unresectable and/or advanced HCC. Taking advantage of the molecular signaling pathways, systemic therapies involving Sorafenib (21), Lenvatinib (22), Nivolumab (23), Regorafenib (24), and Cabozantinib (25) have shown survival benefits in HCC patient cohorts. However, the surgical, systemic, and locoregional therapies currently advocated and in practice for treating HCC are associated with several adverse events summarized in **Table 1**. The ability to systemically treat, albeit partially, the highly chemotherapy resistant HCC tumors and increased understanding of disease pathogenesis are expected to pave way for future therapeutics.

**Table 1:** Adverse events associated with systemic and surgical hepatocellular carcinoma (HCC) treatment approaches.

Therapeutic approach	Target	Adverse events
**SYSTEMIC TREATMENT: 1) Tyrosine Kinase Inhibitors:** Sorafenib, Lenvatinib, Regorafenib, Cabozantinib, Imatinib	PDGFR, FLT3 VEGFR 1-3, FGFR 1-4, PDGFA, Tyrosine kinase receptor, VEGF, MET, AXT, RET, TYRO3, MER- ABL, BCR-ABL, c-KIT	Diarrhea, hand-foot syndrome, hypertension, decreased appetite and weight loss, fatigue, hypothyroidism.
**2) Immune-Checkpoint Inhibitors:** Nivolumab, Pembrolizumab, Durvalumab, Atezolizumab, Tremelimumab, Iplimumab	PD-1 Ab PD-L1 Ab CTLA-4 Ab	Skin rash, fatigue, diarrhea, pruritis, decreased appetite and weight loss, joints and musculoskeletal pain, constipation, dyspnea.
**SURGICAL TREATMENT: 3) Surgical approach:** Liver resection Liver Transplantation	Surgical resection of liver tumor and/or liver transplantation	Pain, fatigue, hypovolemic shock, risk of intrahepatic recurrence, high mortality rate, hepatic failure.
**4) Locoregional therapy:** Radiofrequency Ablation (RFA) Transarterial chemoembolization (TACE) Percutaneous ethanol injection (PEI) Radioembolization Cryotherapy	Tumor targeted therapies	Liver failure, thoracic complications, bile duct injury, intraperitoneal bleeding, hepatic abscess, gastrointestinal perforation, tumor seeding, nausea, pain, fever, loss of appetite, hair loss, low white blood cells and platelets counts, ascites, obstructive jaundice, portal hypertension, radiation pneumonitis, hepatic dysfunction, vascular injury, hematoma, hepatic decompensation.

Since systemic therapies target proliferative and angiogenic pathways involving tyrosine kinases, vascular endothelial growth factor receptor (VEGFR), platelet-derived growth factor receptor (PDGFR)-β tyrosine kinases, fibrosarcoma kinases, etc (26), and HCC is characterized as an immunogenic cancer, greater opportunities can be envisaged for specific and more effective treatment strategies. In general, cancer-associated inflammation, present at different stages of tumorigenesis, contributes to genomic instability, stimulation of angiogenesis, epigenetic modifications, aggressive cancer cell proliferation, enhanced anti-apoptotic pathways, and cancer dissemination (27). Studies in the last two decades have implicated inflammatory pathways in cancer with emphasis on understanding how immune cells impact tumor fate in different stages of disease: early neoplastic transformation, clinically detected tumors, metastatic dissemination, and therapeutic intervention. Despite the significant advances in our understandings of the immunological basis of cancer (28), the immunopathogenesis of HCC remains underexplored.

## Immunopathogenesis of HCC

While the liver is highly tolerogenic and prevents hostile immune responses, organ homeostasis is maintained by natural killer (NK), natural killer T (NKT) cells, γδT cells, Kupffer cells (KCs), etc (29). However, breakdown of this tight regulation by virus infection, alcohol abuse, and lipid accumulation results in chronic inflammation and destruction of hepatocyte and cholangiocyte epithelial cells, leading to cirrhosis (30). Inflammation-associated cellular proliferation, genomic DNA mutations, and reactive oxygen species (ROS) production further enhance malignant transformation (31). In this environment, cancer cells evade immune surveillance and are associated with increased tumor infiltration by immune cells and, amplification of pro-tumorigenic cytokines, etc (32). Several cell-death pathways linked to TNFα, IL-6, NF-κB, STAT3, and JNK, and innate and adaptive immunity are activated in HCC, attesting to the dominant roles of immune mechanisms in hepatocarcinogenesis (30, 33). In particular, innate immune responses involving NK, NKT cells, dendritic cells (DCs), tumor-associated macrophages (TAMs), tumor-associated neutrophils (TANs), myeloid derived suppressor cells (MDSCs), regulatory T cells (Tregs), and cytokines/chemokines derived from these cells form the first-line events in either dampening or promoting tumor initiation and progression within the tumor microenvironment (TME) (34).

In HCC, NK cells are activated by NKT, DC, and KCs, and suppressed by Tregs and hepatic stellate cells (HSCs) (35). However, NK cell numbers are reduced in HCC lesions, with reduced levels of IFNγ and cytotoxic potentials (36), possibly due to hypoxic stress and/or transitory behavior of activating/inhibitory NK receptors. In addition, α-fetoprotein (AFP), MDSCs, and TAMs dampen activating NKG2D receptors and block NK cell cytotoxicities (37). The role of NKT cells, however, remains less understood, with Th2 cytokine-producing tumor-promoting and anti-tumor CD4^+^ NKT cells that accumulate in the TME. Another important component of innate immunity involves DCs that serve as professional antigen-presenting cells (APCs), priming T cells against tumor associated antigens (TAAs) in HCC. However, DCs in patients with HCC remain refractory to high inflammatory cytokine maturation stimuli and show defective antigen presentations due to decreased HLA class-I expressions and a weakened T cell response (38). Furthermore, the frequencies of activated CD83^+^ DCs are lower in HCC livers and absent in tumor nodules, denoting impaired cytotoxic responses.

In parallel with the DC phenotypes, the alternatively activated CD163^+^ M2 TAMs promote tumor initiation, progression, and metastatic malignancy, and are considered as negative prognostic markers associated with low survival rates (39). In HCC, this M2 polarization is sustained by high levels of colony stimulating factor-1 (CSF-1) and reduced innate and adaptive immunity *via* IL4 (40). TAM-derived IL-10 and interactions with MDSCs result in decreased IL-6, IL-12, and MHCII, and increased anti-inflammatory IL-10, TGF-β1, and Foxp3^+^ Treg frequencies to facilitate tumor growth and immune tolerance (41, 42). Similar to TAMs, the recently described TANs recruit macrophages and Tregs to the TME, promoting tumorigenesis and resistance to sorafenib in preclinical studies (43). In patients with HCC, CD66B^+^ neutrophils colocalized with CCL2 and CCL17, infiltrating the liver stroma (44). In experimental models, TANs secrete BMP2 and TGF-β2, trigger miR-301-3p expression in HCC cells, suppress LSAMP and CYLD expressions, and enhance HCC stemness (44). In patient specimens, increased TANs were associated with increased CXCL5 expression and miR-301b-3p levels, decreased LSAMP and CYLD expressions, and nuclear p65 accumulation, collectively contributing to immunosuppression and HCC patient prognosis (45).

The immunosuppressive TME is further elevated by MDSCs, a heterogeneous inhibitory cell population with increased arginase-1, nitric oxide, ROS, and TGF-β activities that promote induction of Tregs (46). While CD14^+^/HLA-DR^–/low^ MDSCs populate HCC livers and block T-cell responses, circulating MDSCs have been negatively correlated with reduced HCC recurrence-free survival (47). Furthermore, MDSCs in the TME suppress IFN-γ production by NKT cells, express Galectin-9 to interact with and induce T-cell apoptosis, and inhibit NK cell cytotoxicity *via* interactions with Nkp30 receptor (48, 49). In HCC, increased intratumoral Treg activity is always associated with defective anti-tumor responses and poor prognosis. Higher frequencies of Tregs were found to be intricately associated with lower CD8^+^ T cell responses, absent tumor encapsulation, and increased tumor vascular invasion (50). A concerted interaction between Amphiregulin (AR)-expressing HCC cells and Tregs triggered mTORC1 expression in Tregs, suppressing CD8^+^ T cell mediated anti-tumor responses. Similarly, inhibiting mTORC1 *via* rapamycin or blocking AR/EGFR signaling using Gefitinib enhanced anti-tumor CD8^+^ T-cell functions, highlighting the importance of Treg-driven processes in HCC TME (51). Similarly, increased accumulation of Tregs in HCC tumors correlated with reduced CD8^+^ T-cell infiltrations and reduced Granzyme A, Granzyme B, and Perforin expressions. Importantly, these events are associated with significantly reduced survival times and increased mortality of HCC patients. Such intratumoral inverse correlations of Tregs and CD8^+^ T-cells also contribute to the prognostic value of HCC patients by facilitating angiogenesis and substantially modulating anti-tumor CD8^+^ T-cell functions (52). An immunosuppressive functional role has also been identified for IL-35 in HCC, a cytokine expressed primarily by Foxp3^+^ Tregs. IL-35 induces conversion of naïve T cells and B cells into Tregs and Bregs, respectively, and is involved in negative regulation of autoimmune diseases (53, 54). Patients with elevated IL-35 were at a higher risk of postoperative recurrence after curative HCC resection and correlated with increased infiltration of a new CD39^+^Foxp3^+^ Treg subset (55). Meta-analysis of 23 studies with a total of 1,279 patients with HCC and 547 healthy controls revealed that a) the frequency of circulating Tregs was 87% higher than in healthy controls and b) intratumoral Treg levels were higher than the surrounding tissue and healthy controls (56).

## Cytokines and Chemokines in HCC

In conjunction with the suppressive functions and escape mechanisms of the immune-cell compartments, several proinflammatory and immunomodulatory Th1 and Th2 cytokines and chemokines define the outcomes of tumorigenesis in HCC (57, 58). The sustained and permissive cytokine and chemokine synthesis in the TME promotes a maladaptive immune response, amplifying dysplastic cellular responses. Immune and epithelial cells within the hepatobiliary system elaborate a range of cytokines with simultaneous expression of receptors.

## Pro- and Anti-Inflammatory Cytokines

In patients with cirrhotic livers, high levels of Kupffer cell derived IL-6 are associated with poor disease prognosis (59). TAMs also utilize IL-6/STAT3 axis to promote expansion of liver cancer stem cells (CSCs) *via* autocrine IL-6 signaling (60). High levels of IL-4 and IL-5 in the TME are also associated with increased HCC metastasis and a polarized Th2 phenotype (61). IL-22, a member of the immunosuppressive IL-10 family, is also elevated in the TME, promoting HCC tumorigenesis, metastasis, and inhibition of apoptosis *via* activation of STAT3 (62). IL-10 itself is upregulated in HCC TME, defining risk of progression after tumor resection (63).

IL-1, IL-18, and IL-36, members of the IL-1 family of the cytokines, are pro-inflammatory and mostly associated with tumor growth. IL-1 induces synthesis of DC-derived CCL22 to recruit immunosuppressive Tregs and further enhance HCC. However, antitumor activity is shown by the presence of IL-36a in HCC, decreased levels of which predict poor prognosis and survival (64). Similarly, IL-37 inhibits HCC growth *via* CD57^+^ NK cells (65), limiting G2/M cell cycle arrest and decreasing cell proliferation (66). The pro-inflammatory cytokines TNF-α and IL-1β are robustly involved in HCC tumor invasion, angiogenesis, and metastasis. IL-1β has been found to increase soluble MHC Class I Polypeptide-Related Sequence A (MICA) thereby blocking NK activity and enhancing HCC (67). TNF-α suppresses anti-tumor CD8^+^ T-cell responses by upregulating macrophage cell surface expressions of the negative co-stimulatory molecules B7H1 or PDL1 (68). IL-1β, while promoting increased synthesis of IL-2, IL-6, and TNF-α, also acts as a tumor growth promoting molecule in conditions of chronic inflammation. TAM-derived IL-1β in the TME is known to drive metastatic potentials of HCC (69). A recent study showed that HCC patients with necrotic tumors harbored significantly higher levels of CD68^+^ TAMs and were associated with elevated levels of serum IL-1β and poor prognosis. Importantly, areas with TAMs showed high expressions of IL-1 receptor, HIF-1α and Vimentin suggesting epithelial mesenchymal transition (EMT). In a Huh-7 xenograft mouse model, the authors showed that IL-1β-induced EMT was mediated through HIF-1α resulting in metastatic lesions (70). Higher levels of TNF-α and IL-1β are also found in tumor-independent areas of tissue metastases (71). In association with TNF-α and IL-1β, increased levels of IL-17A predict poor prognosis (72). IL-17A also induces EMT *via* AKT signaling, promotes invasion/metastasis and HCC cell colonization (73), and increases cell motility by upregulating MMP-2 and MMP-9 and activating NF-κβ (74). IL-17 acts directly on HCC cells, inducing AKT-dependent IL-6/JAK2/STAT3 activation and tumor progression (75). In contrast, increased infiltration of IL-33^+^ cells derived mostly from CD8^+^ T-cells was associated with better prognosis in patients undergoing surgical resection (76). Key cytokines of the IL-2 family, including IL-2 and IL-15, potently stimulate lymphocyte activity and proliferation of cytotoxic T lymphocytes (CTLs) and NK cells. IL-2 enhances CTL activity and IFN-γ production and modulates HCC progression in mice (71). Similarly, an increase in Th1 IL-2 expression is associated with enhanced CD8^+^ T-cell activity, increased IFN-γ production, and improved prognosis (77). IL-15, which positively upregulates proliferation and activation of NK, NK-T, and CD8^+^ T-cells, corrects NK cell dysfunction (78), controls HCC tumorigenesis (79), and promotes tumor-specific CD8^+^ T-cell responses (80).

This cytokine milieu not only regulates developmental and regenerative responses in the liver but also contributes to pathogenesis of hepatic cirrhosis, fibro-inflammation, and HCC. In particular, altered levels of proinflammatory IL-1α, IL-1β, IL-2, TNF-α, and Th2-like IL-4, IL-5, IL-8, and IL-10 cytokines have been associated with HCC phenotypes. In general, the cytokine milieu in HCC is skewed towards an anti-inflammatory over pro-inflammatory environment.

## Role of Chemokines in HCC

Aligned with the pro-tumorigenic roles of cytokines, the chemokines and their receptors promote extravasation of immune cells and migration along a chemotactic gradient towards areas of fibroinflammation. The most relevant chemokine-dependent immunoregulatory pathways in HCC include the CXCL12–CXCR4, CXCL5/8–CXCR2, CCL2–CCR2, and CCL3/5–CCR1/5 axes. The CXCL12-CXCR4 axis represents the most extensively investigated system in HCC, which regulates angiogenesis and promotes tumorigenesis. In liver specimens from HCC patients, CXCL12–CXCR4 signals are more selectively localized to tumors than the adjacent normal or cirrhotic areas (81). In HCC cell lines, this chemokine axis promoted and enhanced the growth, invasion and metastatic potentials (82), and migration of tumor cells (83). Associations of the CXCL12–CXCR4 pathway in supporting metastasis and disease severity have also been demonstrated using HCC cell lines, showing increased MMP2 and MMP9 secretion (84) and decreased 3-year-survival rates in patients (85). Importantly, the CXCL12–CXCR4 axis interacts with MMP10 (86), further supporting tumor development, angiogenesis, and metastasis. The importance of MMPs in early invasion of HCC is further exemplified by the interactions of CXCL12 and CXCR4 with MMP2, MMP7, and MMP9. In this context, the CXCL12–CXCR4 axis provides avenues for development of novel therapeutics (87). In the aforementioned pathway, TGFβ interactions with CXCR4 shift HCC cells towards a mesenchymal phenotype (88) and increase invasiveness when treated with exogenous CXCL12 (89). High levels of CXCR4-expressing OV6+ tumor-initiating cells in HCC patient livers are associated with aggressive pathobiology, increased invasion, metastasis, and poor prognosis (90). Signaling pathways linked to EGF-EGFR in concert with CXCL5 regulate development of HCC (91), while the CXCL5-CXCR2 axis contributes to EMT of HCC cells *via* PI3K/Akt/GSK-3β/Snail signaling (92). CXCL5 also influences the development of an inflammatory TME by regulating the infiltration of MDSCs in HCC tumor sites *via* elaboration of IL-17A in γδ T cells (93). In conjunction with CXCL5, high serum levels of CXCL8 in HCC have been associated with increased tumor burden, aggressiveness, and poor patient prognosis (94). Additionally, epithelial cell derived CXCL8 chemoattracts peritumoral neutrophils and regulates disease progression by stimulating angiogenesis *via* secretion of MMP9 (95) and *via* VEGF–VEGFR2 axis in endothelial cells (96).

Similar to the CXC chemokines, the CC chemokines CCL2 and CCL5 interact with their receptors CCR2 and CCR1/5 respectively and are primarily involved in driving pro-tumorigenic and pro-fibrogenic responses. HSC, hepatocyte, macrophage, and endothelial cell derived CCL2 drives hepatic macrophage infiltrations (97) and provides pro-angiogenic signals *via* VEGF and MMP9 (98). Activation of CCL2–CCR2 also promotes migration, invasion, epithelial-mesenchymal transition, and metastasis of HCC *via* endothelial progenitor cells (99). Correspondingly, CCL5 promotes fibrogenic responses *via* resident Kupffer cells, bone marrow-derived macrophages, and HSCs (100) necessary for development of HCC. Investigations into other CCL chemokines CCL19, CCL20, and CCL21 showed specific upregulation of CCL20 in HCC tissues, together with increased expressions of the cognate receptor CCR6 (101). The authors demonstrated that CCL20-CCR6 axis regulates tumorigenicity in HCC, with increased CCL20 and CCR6 expressions in grade III tumors. Elevated expressions of CCR6 also correlate with formation of pseudopodia in HCC cell lines, increased metastasis, and poor survival in patients (102). Recent studies have also identified Fractalkine–CX3CR1 interactions in HCC cell cycle and CX3CL1 dependent cytotoxic T cell, IL-2, and IFN-γ responses that block tumor development (103).

In summary, the immune cells, soluble effector molecules and the chemokine receptors have been a subject of intense research and investigations as potential therapeutic targets to treat the chronic inflammatory states in HCC.

## Targeting Immunity in HCC: Current Strategies, Limitations, and New Mechanisms

As discussed above, the complex interplay of the immune cells with soluble effector molecules in chronic inflammatory states of HCC alters the immune system, either suppressing or facilitating tumor growth. Harnessing these multimodal mechanisms *via* immunotherapeutics is therefore expected to be beneficial in early and advanced stages of HCC. Utilizing the differential responses, systemic therapies to perturb VEGF-dependent angiogenesis, WNT, PI3K/AKT/mTOR, AMPK, and c-MET pathways in the TME are either approved or in clinical trials (104). However, Sorafenib, an oral tyrosine kinase inhibitor (TKI), remains the only FDA-approved treatment with survival benefits for HCC. It inhibits VEGFR, Raf-1, B-Raf, platelet-derived growth factor receptor (PDGFR), c-KIT receptor, and p38 signaling pathways involved in angiogenesis and tumor proliferation (105). Notwithstanding the survival benefits, therapeutic efficacy of Sorafenib is limited, with patients experiencing severe adverse effects and disease progression; prognosis is poor in patients discontinuing Sorafenib, with no additional available therapies (106). Similarly, Lenvatinib is a first-line TKI for unresectable HCC currently in clinical trials (107). Second-line therapies for advanced HCC that are intolerant to first-line treatment include Regorafenib, Cabozantinib, Sunitinib, Linifanib, Brivanib, Tivantinib, Donafenib, etc. which target tyrosine kinases, HGF-MET axis, and related pathways (108). Though many of these newer therapies show improved survival with robust and durable responses, development of drug resistance, severe adverse events, and cytostatic properties limit therapeutic benefits and patient acceptability. In this context, several indirect and direct immunotherapies that target adaptive and innate immune cells have been developed. The immune checkpoint inhibitors (ICIs) block T cell activation and promote T cell exhaustion by primarily targeting either CTLA-4 (cytotoxic T lymphocyte antigen-4; CD152) or PD-1/PD-L1, some currently approved or under clinical trials for HCC (109). Anti-PD-1 ICIs (Nivolumab, Pembrolizumab, Tislelizumab, Camrelizumab, Cemiplimab, Sintilimab) block the co-inhibitory receptor PD-1 on T cells, activating antitumor T cell responses, durable response, and improved survival. Alternatively, anti-PD-L1 ICIs (Durvalumab, Atezolizumab, Avelumab) target increased PD-L1 expression on DCs, macrophages, T and B cells, and tumor and endothelial cells. Mono or combination therapies have demonstrated reasonable response rates, improving progression-free survival (110). Similarly, in an immunosuppressive environment, CTLA-4 inhibits T cell activation, promotes Treg differentiation, and deregulates antigen-presenting functions of DCs (111). The anti-CTLA-4 ICIs (Tremelimumab, Ipilimumab) enhance anti-tumor immunity by reducing Treg frequencies, increasing activation threshold, and preventing anergy of T cells (112). Adoptive Cell Therapies (ACTs) that form the other arm of direct immunotherapy target HCC *via ex vivo* genetic modifications of autologous immune cells (113).

It is known that HCC-associated inflammation contributes to genomic instability, epigenetic modification, induction of cancer cell proliferation, enhancement of anti-apoptotic pathways, stimulation of angiogenesis, and eventually, cancer dissemination (30). Since immune cells are an essential player of HCC-related inflammation, efforts have focused on understanding how these cells impact tumor fate in different stages of disease: early neoplastic transformation, clinically detected tumors, metastatic dissemination, and therapeutic intervention (28). The aforementioned approaches on modulating the immune environment to treat HCC demonstrate limited feasibility of available therapies and offer opportunities for mechanistic explorations and development of effective HCC treatment measures. Monotherapy with ICIs, ACT, etc. have largely failed to meet the primary clinical endpoints of antitumor responses and decreased tumor size (114). Development of resistance, heterogeneity of tumors, circumvention of inhibitory mechanisms that prevent anti-tumor responses, altered TME, hypervascularity, hypoxia, severe adverse events, potential transplant rejection, etc. further complicate the use of ICIs in effectively managing HCC (115). Furthermore, these strategies mostly temper a singular population of cells and oversimplify the complex and multifaceted immune responses in the TME. As the current immunotherapies rely mostly on modulating adaptive immune responses, deciphering novel mechanisms involving innate immunity can improve therapeutic efficacy and reduce HCC burden. Newer treatment protocols may therefore take advantage of combining novel therapeutic agents with existing first and second-line therapies. One such area of mechanistic investigation and approach garnering significant attention is modulation of the multiple components of the Complement cascade (“CC” or “C”). CC is a critical and integral arm of the innate immune response involving the Complement system (C) that not only enhances the effects of antibodies and eliminates cellular debris, foreign intruders, and dead cells but also tightly regulates liver injury, inflammation, and regenerative responses (116).

## Complement System: Regulation of Immune Responses

**Figure 1:**
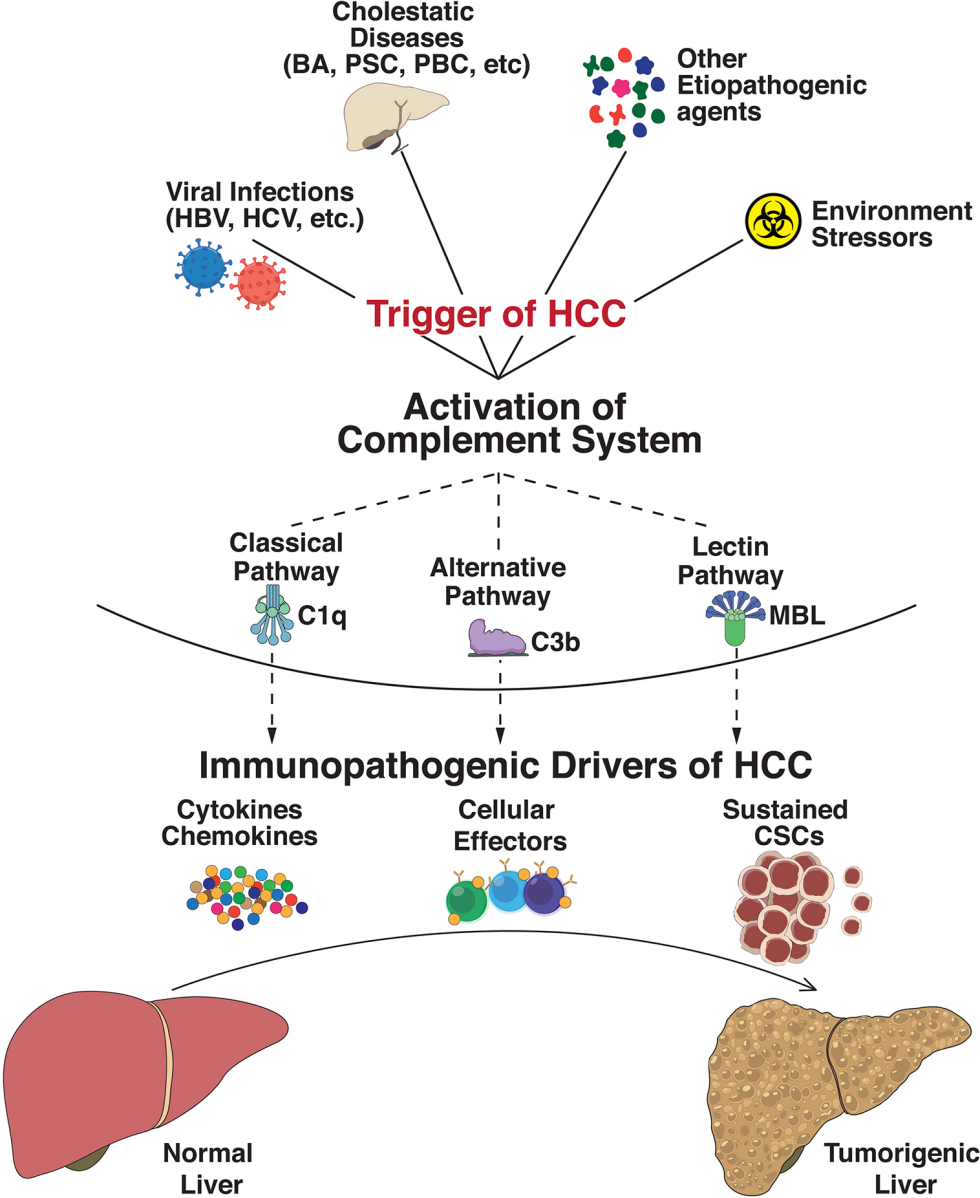
Immunopathogenic complement activation regulates progression to hepatocellular carcinoma. Exposure of the hepatic milieu to several triggers linked either to viral infections (Hepatitis B virus, Hepatitis C virus, etc.), severe obstructive and cholestatic diseases (Biliary atresia, Primary Sclerosing Cholangitis, Primary Biliary Cirrhosis, etc.), environmental stressors/toxin exposures (polychlorinated biphenyls, arsenic, androgenic steroids, etc.), and other etiopathogenic agents (aflatoxins, oral contraceptives, vinyl chloride, etc.) dictate the evolution of hepatocellular carcinoma (HCC). These triggers activate the innate immune complement cascade *via* classical (involving C1q complex), alternative (C3b-dependent activation), or lectin (triggered by carbohydrates) pathways. Abnormal activation of these complement pathways modulates functional effects of intrahepatic immune and epithelial cell compartments and disseminates significant perturbation of effector innate and adaptive cells, cytokine and chemokine expressions, and sustained cancer stem cell (CSC) activities. The collective net result of these processes defines the progression of HCC tumorigenesis.

The complement system is an integral part of the innate immune response with abilities to discriminate self from non-self, and rapidly eliminate invasive pathogens while causing minimal injury to the host (117). It is an intricate system with broader functions in immune surveillance and homeostasis, controlled through a balance of activating and regulatory proteins (118). Complement activation occurs *via* three major pathways: classical pathway (CP), lectin pathway (LP), and alternative pathway (AP), which merge into a common terminal pathway to activate C3. CP and LP are activated by antibodies and other pattern recognition molecules whereas AP is continually activated in plasma through a process called tick-over *via* continuous formation of C3b (119). Activation of C3 leads to formation of C5 convertase which cleaves component 5 (C5) into C5a and C5b. C5b then binds to C6 and C7 to form the C5b–C6–C7 complex. This complex interacts with C8 and C9 to form the membrane attack complex (MAC), resulting in antibody-mediated complement-dependent cytotoxicity (CDC) when inserted into a membrane. These activated proteins can then be deposited on cell surfaces or released into body fluids to interact with specific receptors, leading to lysis of foreign cells *via* cytoplasmic swelling and rupture of cell membranes, which are classical characteristics of necrosis (120). However, recent studies provide new perspectives on the immunosuppressive functions of complement components. Over the last decade, studies have demonstrated that these complement components could contribute to regulating the functions and tumor-suppressing immune responses (121). Recent findings suggest an insidious relationship between complement and cancer, tumorigenic competency of the complement system, cellular proliferation, and regeneration. Complement principally plays a protective role against tumor formation in humans (122) while also contributing to a large variety of divergent inflammatory and immune processes (123). Since HCC has underlying origins of chronic and ectopic inflammatory states, premature complement activation can be envisaged as a potential driver of onco-inflammatory processes. Indeed, altered or enhanced complement activation underlies a wide spectrum of inflammatory diseases including asthma (124), kidney and cardiac diseases (125, 126), multiple sclerosis (127), and rheumatoid arthritis (128). In addition, complement regulates several key biological processes including liver injury and regeneration (116), cellular proliferation (129), angiogenesis (130), epithelial mesenchymal transition (131), and metastasis (132). An overview of the etiopathogenic events in HCC triggering complement activation is shown in **Figure 1**.

## Complement Proteins in HCC

**Figure 2:**
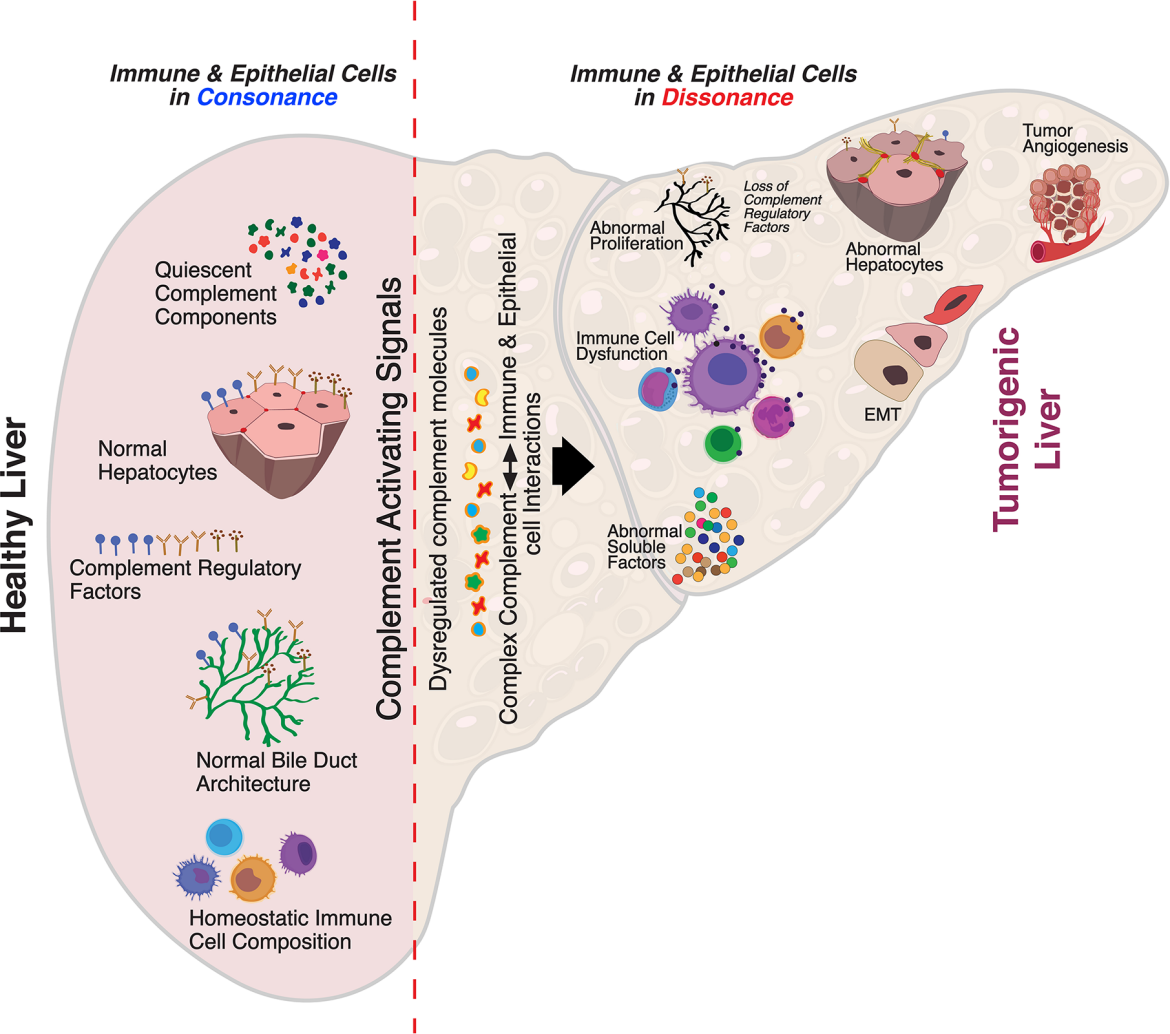
Aberrant complement activation: a driver for disease progression in hepatocellular carcinoma. A schematic of the liver microenvironment depicting the transition of a healthy immunologically quiescent intrahepatic microenvironment to dysregulated immune status following activation cues to the complement system. In a healthy liver, immune and epithelial cells function in synergy to preserve normal architecture of bile ducts, quiescence of complement molecules, and homeostasis of immune cells. Complement activating disease-triggering signals orchestrate the evolution of dysregulated complement molecules (increased C3, C5, etc) and altered complement regulatory factors (CFH, etc). These acute and/or chronic sustenance of dysregulated complement molecules and their complex interaction with the immune and epithelial cell compartments drive the progression of hepatocellular carcinoma (HCC). Loss of complement regulatory factors and divergent activation of the complement system leads to abnormal hepatocyte architecture, deranged cellular and effector functions, and reactive bile duct profiles. Cumulatively, these events lead to epithelial to mesenchymal transition (EMT) and tumor angiogenesis which worsen the disease, resulting in poor clinical outcomes and death.

Despite substantial research on the role of inflammatory cells and their immunosurveillance within the TME, little attention has been given to the tumor propagating properties of the complement cascade. Although increased levels of complement proteins in malignant tumors promote proliferative tumorigenesis, the exact role of complement in HCC remains unclear. The relevance of the complement system is further underscored by its ability to principally regulate the cellular and molecular events in HCC including TAMs, TANs, Tregs, MDSCs, DCs, NK cells, and cytokine (IL-1, IL-2, TNF-α, IL-4, IL-10, etc.) and chemokine axes (CXCL12–CXCR4, CCL2–CCR2, etc). Complement activation therefore can promote HCC *via* enhanced angiogenesis, protection of tumor cells from immunosurveillance, increased mitogenic signaling, activation of anti-apoptotic mechanisms and aberrant cell proliferation, invasion, and migration (133). It is only recently that the complement proteins have garnered interests in cancer through immunosuppression and their roles in promoting HCC are being discovered. Complement activation has also been linked with the development and spread of several cancers, raising the possibility that impaired complement regulation could be a risk factor for oncogenesis (119). In this context, recent work by Mittal et al., discussed the ability of the immune system to act against tumor progression in an “immune-editing process” composed of three distinct phases: elimination, equilibrium, and escape (134). The authors showed that the immunological responses were able to prevent tumor progression in elimination & equilibrium phase whereas the acquired adaptations of malignant cells and the host immune system allowed for expansion of the tumor cell population during the escape phase. The complement system, an integral component of the antitumor immune response, acts as an intrinsic effector mechanism to form a functional bridge between the innate and the adaptive immune system thereby promoting or suppressing tumor development. Complement activation within the liver may therefore contribute to the development of HCC by several mechanisms, for example, *via* activation of NF-κB in Kupffer cells and STAT3 in hepatocytes. While these events facilitate recovery of liver after acute injury, the sustained chronic activation promotes hepatocyte proliferation and development of HCC (135–137). **Figure 2** depicts the loss of homeostasis resulting in dysregulated complement activation, immune responses and biological processes promoting amplified hepatic oncogenic responses in HCC. In the following section, the roles of several components of the complement system in the etiology, pathogenesis, and therapeutic modulation of HCC are discussed.

### Complement Factor H

CFH is a soluble complement protein expressed constitutively in the liver (138) by epithelial (139) and endothelial (140) cells, platelets (141), etc. CFH regulates the activation of AP by accelerating the decay of AP C3 convertase and inactivating C3b (142). A recent study using CFH-deficient mice demonstrated the importance of CFH in controlling hepatobiliary complement activation, absence of which resulted in chronic inflammation and development of HCC (119). CFH-deficient mice showed extensive complement activation and hepatocellular inflammation as early as 3 months of age and developed liver steatosis and chronic hepatic injury followed by HCC in >50% of mice by 15 months of age, confirming the role of activated AP in HCC (119). The tumor-suppressive effects of CFH in liver carcinogenesis were further confirmed by analyzing gene expression and methylation profiles in patients with HCC (143). Bioinformatic analysis by Laskowski et al., revealed that patients with mutations in the CFH gene were reported to have a median disease/progression-free time of less than a year compared to almost 2 years for those without mutations (119). In addition to rendering the hepatic environment susceptible to carcinogenesis, Seol et al. reported the co-operativity of CFH and complement component C7 in maintaining the stemness and replication competency of tumor-initiating hepatocarcinoma cells (144). Using tumor-sphere cultures, the authors showed that absence of C7 and CFH abrogated tumor-sphere formation with restored stem cell proliferation in liver cells that overexpress these components. The ability of CFH and C7 to maintain cancer cell stemness was mediated through the induction of Late SV-40 factor (LSF-1) which plays a prominent oncogenic role in HCC and is overexpressed in >90% of patients with HCC (145). Inhibition of LSF significantly attenuated metastasis of HCC in nude mice while overexpression promoted aggressiveness and angiogenic and metastatic potentials of HCC tumors. It is important to note that LSF regulates a string of proteins involved in hepatocarcinogenesis, including osteopontin (OPN) (146). OPN sequesters CFH on the surface of the tumor cells and hinders the formation of membrane attack complex (MAC), effectively preventing complement-mediated lysis and enabling tumor cells to escape immune surveillance (146). Several recent studies have also investigated the role of Complement factor H-related 3 (CFHR3) in HCC, which until recently, remained unexplored. CFHR3, a member of the human factor H family, exhibited significantly lower mRNA and protein levels in HCC tumor tissue. Studies by Liu et al. showed overexpression of CFHR3 blocked cellular proliferation and viability, and enhanced apoptosis (147). In patients with HCC, differential expression levels of CFHR3 correlated with better prognosis (148). Gene enrichment analysis showed decreased CFHR3 expressions with pathways upregulated in tumorigenesis including regulation of cell activation cycle and WNT and NOTCH signaling pathways. Searching for novel prognostic biomarkers of HCC, Pan et al. identified a panel of 10 differentially expressed genes between cohorts of patients with high and low infiltrations of immune and stromal cells (149). The 10-gene signature predicted a favorable outcome of overall survival in HCC patients. The presence of CFHR3 in conjunction with other markers in the TME may therefore serve as a prognostic predictor for targeted therapeutics in HCC. Similarly, RNASeq data analysis of HCC patients identified 17 genes with significant effect on HCC prognosis (150). Of these, a set of seven genes that contained CFHR1 defined a clinical prognostic signature that predicts the survival of HCC patients. Collectively, these data point towards treatment options that enhance CFH/CFHR levels either by gene therapies or by CFH reconstitution to lower tumor burden in HCC.

### CD59

CD59 is another mCRP that is involved in restricting initiation and progression of complement activation on cell surfaces. In general, downregulation of CD59 promotes the activation of complement‐mediated cell lysis while increased expression can confer resistance to cancer cells (151). Low levels of CD59 are also linked to several autoimmune diseases including rheumatic diseases (152), autoimmune thrombocytopenia (153), diabetes (154), and multiple sclerosis (155). In some instances, increased expression of CD59 correlates with overall decreased survival rates in patients with colorectal cancer (156), prostate cancer (157), and B cell lymphoma (158) while low CD59 expression in breast tumors correlates with increased invasiveness and poor survival (159). Regardless of the spectrum of expression, extreme variations in the levels of CD59 result in pathologic outcomes of oncogenesis or cancer progression (160). Recent studies have linked CD59 to pathogenesis of HCC by prevention of complement mediated apoptosis. Abdel-Latif et al. showed increased mRNA and protein expressions of CD59 in a DEN-induced rat model of HCC that promoted enhanced tumor growth (161). In rats with HCC, increased levels of serum CD59 were not linked to phospholipase D (PLD)-dependent cleavage of CD59 (162) but rather to inflammation driven shedding from membrane lipid rafts (163, 164). Treatment with CoenzymeQ10 resulted in decreased CD59 and proinflammatory responses, providing protection against HCC. Using ChIP assays to study the role of the Hepatitis B virus (HBV) X protein (HBx) in HCC development, Shan et al. reported the upregulation of CD59 levels and protection from complement-dependent cytotoxicity (CDC) (165). siRNA mediated downregulation of CD59 sensitized the HBx-positive tumor cells and rendered them susceptible to CDC, suggesting new therapeutic avenues in HBV–HCC patients (165). Recent work by Lan et al. has shown that CD59 can function as a potential oncogenic driver in HCC and metastasis. Liver specimens from HCC patients showed high expressions of CD59 that correlated with poor overall and disease-free survival. Mechanistically, the authors showed that loss of CD59 impaired *in vitro* and *in vivo* tumorigenic and metastatic capacities *via* excessive Smad7 formation and abolishment of Smad2/3 phosphorylation. Therefore, CD59 facilitates HCC pathogenesis *via* suppression of CDC and modulation of TGF-β signaling; it may serve as an effective prognostic biomarker and potential therapeutic target in HCC (166).

### C1q

C1q forms the recognition element of complement component C1 as a complex with the proteases C1r and C1s involved in activation of the classical pathway (167). The C1q complex is not only involved in recognition of complement activating elements, but also in regulation of autoimmune diseases (168) and in prostate cancer *via* the activation of tumor suppressor molecule WOX1 (WW-domain containing oxidoreductase) (169). Unlike other complement proteins, C1q is synthesized by several cell types relevant to the pathophysiology of oncogenesis, including epithelial and mesenchymal cells (170), monocytes/macrophages (171), dendritic cells (172), fibroblasts (173), and endothelial cells (174). In addition, the human hepatoma-derived cell line HepG2 secretes functional complement proteins C1r, C1s, C2, C3, C4, C5, etc. (175). Emerging data shows the involvement of C1q in progression and survival of cancer cells. Similar to requirements of increased expressions in preventing autoimmune diseases (176), C1q sustains WOX1 in blocking cell proliferation and hyperplasia in prostate cancer (169). C1q interacts with cell surface binding proteins cC1q-R and gC1q-R (177) that show divergent roles in cancer, with cC1q-R showing tumor suppressive activity (178) and gC1q-R promoting tumor cell progression and metastasis (179). Earlier studies have shown measurement of C1q-binding serum factors as a useful method in monitoring tumor growth in experimental animals (180) and enhanced C1q inhibition activity in sera of patients with HCC (181). In a similar approach, C1q solid phase assays were used to detect hepatitis B surface antigen (HBsAg) in HBsAg+ and HBsAg– patients with primary HCC (182) and to detect increased levels of CICs in HCC patients (183). Similar to C1q, Yao et al. showed the ability of HCV core/gC1qR interactions to suppress T cell immune responses, resulting in persistent infection (184). Takeuchi et al. further showed that C1qTNF6 is overexpressed in HCC tissue specimens and contributes to tumor angiogenesis by activating Akt pathway (185). A direct functional role for C1q in the tumor microenvironment was demonstrated in wild-type mice that showed early C1q deposition, high vascular density, and increased lung metastasis compared to C1qa-deficient mice. Results showed that C1q directly regulates complement activation, cancer cell adhesion, migration, and proliferation (186). Recent seminal work by Lee et al. showed that the collagen-like portion of C1q induces activation and upregulation of discoidin domain receptor 1 (DDR1), a collagen receptor, resulting in enhanced migration and invasion of HepG2 cells. C1q induced activation of MAPKs and PI3K/Akt signaling, and increased MMP2 and MMP9 expressions, strongly suggesting C1q–DDR1 interactions in the progression of HCC (187). In this context, MMP2 and MMP9 have been shown to regulate the migrative and invasive capacities of HepG2 cells (188). Independent of these primary functions of C1q, pioneering work by Ho et al. showed that C1q released from macrophages provided an unconventional signal that activated the β–catenin pathway and induced expansion and de-differentiation of periportal hepatic progenitor cells (HPCs). Treatment with C1q inhibitors blocked the β–catenin pathway and expansion of liver tumors, identifying a hitherto unknown pathway of hepatocarcinogenesis (189). Recognition of these novel regulatory pathways for C1q is expected to further expedite mechanistic understandings and design of new approaches for HCC treatment.

### Complement Component C2 (C2)

Complement C2 is an important component of the complement cascade necessary for the formation of C3 convertase, a serine protease significantly associated with HCC. Analogous to CFH, Ning et al. reported that higher expressions of C2 were associated significantly with better prognosis in HCC patients, implicating a protective role for C2. Investigations showed that C2 influenced HCC prognosis *via* several mechanisms, including higher levels of tumor infiltrating CD4+ T and M0 macrophage cells in HCC patients with higher and lower levels of C2, respectively (190). These findings are important considering the association of high mortality rates and reduced survival time in HCC patients with loss of CD4^+^ cytotoxic T cells (191) and M2 polarization of TAMs that promote tumorigenesis, angiogenesis, and metastasis (40). The ability of C2 to suppress HCC and regulate multiple biological processes was supported by the identification of pathways linked to cell cycle, complement and coagulation cascades, AMPK, and PPAR signaling pathways in patients with elevated C2 expressions. The importance of C2 is further exemplified by associations of single nucleotide polymorphisms (SNP) with disease severity of HCC. While C2 SNP rs9267665 is associated with increased risk of developing HCC, the SNP rs10947223 affords protection from HCC (192) (193). Higher expressions of C2 are therefore beneficial for HCC prognosis and modulating complement C2 levels can afford novel therapeutic avenues.

### Complement Component C3 (C3)

C3, the central component of the complement system is also activated in the milieu of oncogenic development. Under normal physiologic and homeostatic conditions, C3 is primarily produced by hepatocytes and restricted mostly to the extracellular space. Several lines of evidence have now confirmed that C3 is generated locally as well as intracellularly by almost all cell types including myeloid, lymphocytic, fibroblastic, and epithelial cells (194). Within the TME, C3 is produced either systematically by tumors (195), or by tumor infiltrating CD8^+^ T cells (196). This tumor cell-derived C3 imparts an immunosuppressive TME by regulating the activity of TAMs *via* C3a–C3AR–PI3Kγ signaling and suppressing antitumor responses (197). Increased activation of intracellular C3 significantly suppressed anti-tumor activity of CD8^+^ T cells, enhanced T-cell exhaustion, promoted an environment rich in immunosuppressive M2 macrophages, and provided resistance to cell lines against anti-PD-L1 treatment. However, blocking tumor cell derived complement C3 enhanced antitumor functions by enhancing the efficacy of anti–PD-L1 treatment, suggesting C3 in combination with ICIs as a potential target for HCC therapy. In HCC, hepatic stellate cells (HSCs) promote complement C3 mediated immunosuppression by restricting proliferation and enhancing T-cell apoptosis, decreasing DC maturation, and amplifying expansion of MDSCs (198). Blocking or modulating C3 functions may not only augment existing treatments, but also dampen cellular responses promoting fibrosis. Equally important is HSC-driven maturation of DCs into MDSCs, a function critically dependent on the presence of C3; complement C3-deficient HSCs, however, fail to induce MDSCs. This immunosuppressive function was linked to HSC derived factor B and factor D, resulting in C3 cleavage to iC3b and C3d; addition of iC3b also promoted differentiation of immunosuppressive MDSCs (199). In HCC, MDSCs promote angiogenesis and immunosuppression. Several clinical studies show the translational importance of MDSC activities (200), providing rationale for future studies that simultaneously target C3 and MDSCs. Furthermore, levels of immunosuppressive iNOS, Arg-1, and IL-4Ra were augmented in HSC-induced MDSCs *via* activation of the COX2–PGE_2_–EP4 signaling pathway. Inhibition of PGE_2_ blocked HCC growth by decreasing HSC-induced MDSC accumulation. Complement C3 and PGE_2_ may also participate in M2 polarization of macrophages in the TME to enhance anti-inflammatory effects (201). The biological roles of C3, however, precede its identification in serum of patients with HCC of HCV origin by MALDI-TOF and complement component C4 as potential biomarkers (202). In addition, other studies identified the diagnostic roles of serum complement C3a in HCC. Using proteomics analysis, Leung et al. identified lower levels of C3a C-terminal truncated fragment in HCC serum *via* SELDI technology, suggesting its value as a serum biomarker for HCC (203). Using a related technology of SELDI-TOF MS analysis, Lee et al. identified complement C3a to be specifically and differentially elevated in patients with chronic hepatitis C and HCV-related HCC (204). These findings were further corroborated by a recent study by Kanmura et al. who aimed to identify novel diagnostic markers for HCC using ProteinChip SELDI system (205). Results showed that a combination of complement C3a fragment, AFP, and des-gamma-carboxy prothrombin (DCP) resulted in 98% positive identification rate. These recent advances in complement-based diagnostic markers are of clinical significance since AFP is the only diagnostic marker indicative of HCC, albeit in about 60% of cases. Complement C3, therefore, plays a central role in biological functions and as a potential biomarker and therapeutic modality.

### Complement Component C5 (C5)

Complement component C5 forms the terminal and an integral component of the complement cascade (206) and is expressed by and interacts with C5AR1 on several cells including lymphocytes, macrophages/monocytes, myeloid cells, hematopoietic stem cells, epithelial cells, and more importantly cells undergoing oncogenic transformations (207). In the context of cancer, the C5-C5AR1 signaling modulates proliferative, anti-apoptotic and prosurvival pathways (208). Upon activation, complement component C5 generates C5a, an anaphylatoxin and a leukocyte chemoattractant, and plays a crucial role in TME by promoting metastasis of cancer cells. In patients with chronic HBV infection, serum complement component C5a is upregulated, predisposing the patients to develop HCC. Tumor cells from HCC patients as well as HCC cell lines show significant upregulation of the complement C5a receptor, C5AR1 (209). Activation of C5aR by C5a enhances the dissemination of circulating tumor cells (CTCs) in HCC *via* upregulation of INHBA/Activin and induction of EMT/MMP by phosphorylation of Smad2/3 (210). Hu et al. demonstrated that C5a ligation of C5aR resulted in activation of the ERK1/2 pathway and induced EMT by increasing Snail expression and downregulating E-cadherin and Claudin-1 expressions (209). While not much of the pathobiological role of complement C5/C5a–C5AR1 axis is known in HCC, its ability to critically influence and control signaling processes relevant to HCC is largely evident from several studies. Activation of the C5a-C5AR1 axis mediates tumorigenic polarization of TAMs *via* NF-κB pathway in metastatic liver lesions (211), while suppressing IL-12 production (212) and promoting immunosuppressive TME *via* C5aR1^+^ macrophages (213). Increased C5ar1 expression also facilitates recruitment of other myeloid cells like neutrophils *via* IL-1 production (214) and leukotriene B4 (LTB4) (215), while C5a stimulates neutrophil derived tissue factor (TF) synthesis, enhancing tumor growth and metastasis formation (216). As a potent chemoattractant of MDSCs to primary tumors (217), C5a can augment disease severity in HCC by suppressing CD8^+^ T cell function *via* immunosuppressive MDSCs (218). In Lewis lung cancer model, blockade of C5aR reduced MDSCs and inhibited tumor growth (219). Additionally, signaling *via* C5a–C5aR promotes Treg expansion and suppresses T cell responses in breast cancer metastasis (220), and increases expression of MCP-1, IL-10, Arg-1, and TGF-β1 in colon cancer tumor metastasis (221). Progressive HCC is typified by EMT with matrix metalloproteinases (MMPs) expressed in the TME predominating an important role in this process. C5a expressed by tumor cells triggers expression of MMPs, enhancing tumor invasiveness, release of pro-angiogenic factors, and cell migration (222). Collectively, complement C5a–C5AR1 axis plays a central role as a regulator of innate and adaptive immunity in the TME and a plausible target for development of novel therapeutics for HCC.

### Complement Receptor 1 (CR1)

Complement receptor 1 (CR1, CD35) is a glycoprotein expressed either on the membrane or in soluble form on erythrocytes, DCs, monocytes, neutrophils, and B and T cells (223). CR1 inhibits both classical and alternative pathways of complement activation by binding C1q, cleaved C3b and C4b, MBL-2, collectins, and ficolins (224–226) on altered cell surfaces to prevent the formation of terminal membrane attack complex (MAC). Erythrocyte CR1 (E-CR1) is important for processing and removal of circulating immune complexes (CICs) to prevent tissue deposition (227). In HCC, serum CIC levels are abnormally high, with pathological implications (183). The ability of CR1 to bind CICs is particularly important in HCC, with underlying viral etiologies where free and IC-associated HCV binds to E-CR1, differentially driving HCV-IC related features of the disease (228). Kanto et al. showed an inverse correlation between low E-CR1 levels and higher C3d immune complexes. Incidentally, low E-CR1 correlated with severe liver inflammation, cirrhosis, and HCC than those with mild inflammation, demonstrating the relationship between IC and HCV disease severity (229). Similarly, low E-CR1 and high levels of IC were observed in patients with chronic hepatitis and liver cirrhosis (230), emphasizing the importance of defective CIC clearance by altered CR1 functions. A recent study by Luo et al. analyzed genetic polymorphisms and found that two SNPs in CR1 gene (rs3811381 and rs2274567) can potentially predispose subgroups of males, alcohol drinkers, and nonsmokers to HBV-HCC and HBV-chronic hepatitis B risks, while decreasing the risk to HBV-liver cirrhosis in females (231). In contrast, soluble sCR1 levels are increased in liver cirrhosis, end-stage renal failure, and hematologic malignancies (232). In addition, increased levels of sCR1 have been found in patients with increasing grades of cirrhosis and decreased liver functions (233). Since sCR1 levels are elevated in these inflammatory conditions, it is envisaged to play important regulatory and anti-inflammatory roles and act as a potential therapeutic target. Preclinical efficacies of a recombinant form of sCR1 with binding sites for C3b and C4b have been assessed in autoimmune and inflammatory disorders with a potential clinical use in HCC (234).

### Mannose Binding Lectin (MBL)

Mannose-binding lectin is an important component involved in the lectin pathway of complement activation (235). MBL functions as a pattern recognition molecule in senescent fibroblast sensing (236), autoimmunity (237), and apoptotic/necrotic cell clearing (238). Further, MBL regulates anti-cancer immunity, plays a diverse role in TME, and contributes to either development or inhibition of tumor growth, depending on the type of cancer (239). MBL2 is primarily produced and secreted by liver cells with significantly elevated levels found in HCC and in HepG2 cell lines (240). Proteomic analysis of serum in patients with pancreatic cancer showed increased levels of MBL2 as a marker of potential diagnostic value (241). Using seven publicly available protein and gene databases, Awan et al. performed enrichment analysis and identified 6 proteins, including MBL2, that defined the biomarkers of HCC (242). This study also identified MBL2 to be a target of 11 circulating and 48 deregulated miRNAs, suggesting MBL2 as a strong candidate for biomarker discovery in HCC (242). Exploring the little-known roles of MBL in TME, Li et al. showed that the genetic loss of MBL in a murine model of HCC triggered enhanced tumorigenesis compared to wild-type mice (243). MBL-deficient mice showed increased accumulation of MDSCs, Treg induction, impaired CD8^+^ T cell function, COX-2 expression, and PGE_2_ production in tumor tissues. Mechanistically, MBL inhibited hepatic stellate cell activation *via* downregulation of ERK/COX-2/PGE_2_ signaling pathway. Restoring MBL in these mice significantly reduced HCC progression by inhibiting HSC activation, suggesting MBL to be a potential therapeutic option for HCC. Jalal et al. explored circulating liver-derived lectins and found elevated serum binding activities of ficolin-2 and MBL as potential biomarkers of HCC development in chronic HCV infection (244). Interestingly, dysregulation of miRNAs has been associated with progressive HCC. miR-942-3p was found to be increased in HCC tissue and cell lines and was associated with tumor metastasis and poor patient prognosis. In cell lines, ectopic expression of miR-942-3p resulted in enhanced proliferation and invasiveness while restoration of MBL2 blocked progression of HCC and tumorigenic responses (245). Several studies have also investigated associations of genetic polymorphisms in MBL and altered functionality with HCC. MBL rs7096206 polymorphism was associated with polymorphisms in VDR/VEGF and IL-18 which collectively conferred susceptibility to HCC in Asian populations (246), while MBL2 polymorphisms tended to influence the outcomes of HCC susceptibility, progressive tumor development, and clinical outcomes in patients infected with HBV (247). Mutations in MBL2 are also proposed to predispose patients to elevated HCC risk with significantly reduced serum MBL2 and increased IL-6 and IL-1β levels in HCC (248). Similar analysis in HBV-related cirrhotic patients with HCC suggested that MBL2 SNP rs11003123 was a potential risk factor for HCC development in the Chinese population (249). The importance of MBL gene polymorphisms in progressive forms of severe hepatitis B and liver cirrhosis was further supported by a larger meta-analysis study (250). While some patients with chronic hepatitis B or C infection showed lowered levels of MBL (251), studies by Segat et al. showed no significant associations of MBL2 and MASP2 polymorphisms with either HBV/HCV infection dependent HCC or for HCC alone (252).

### Mannose-Binding Lectin (MBL)-Associated Serine Protease-2 (MASP-2)

The MBL-associated protease MASP-2 predominantly promotes activation of the lectin complement pathway. While MASP-2 and lectin pathway components are highly conserved in immune defenses, loss of MASP-2 regulates infectious or autoimmune diseases, immunodeficiency of which are significantly associated with pyogenic bacterial infections, inflammatory lung disease, and autoimmunity (253). In oncogenic environments, increased levels of serum MASP and related lectin pathway molecules have been found to be associated with poor overall survival, disease progression, recurrence, and worse disease prognosis in patients with colorectal (254–256), ovarian (257), and cervical (258) cancers. Similar increases in MASP-2 protein were also associated with advanced clinical stages and nodal metastasis in esophageal squamous cell carcinoma (259). In agreement with these findings, serum MASP-2 levels were higher in pediatric patients with acute lymphoblastic leukemia, non-Hodgkin lymphoma, and CNS tumors (260). However, significant variations between pediatric and adult patients have been documented. In contrast to the severe disease pathogenesis defined by elevated levels of MASP-2, MASP-2 deficiency in leukemic children on chemotherapy was associated with increased risk of febrile neutropenia (FN), antimicrobial therapy, and prolonged duration of hospitalization (261). Similarly, higher serum levels of MASP-2 were associated with impaired event-free survival in pediatric patients with lymphoma (262). Schlapbach et al. further showed that MASP-2 deficient children had significantly increased risk of developing FN in children on chemotherapy (263). These studies show MASP-2 deficiency as a potential risk factor for infections. In the context of HCC, analysis of patient secretomes derived from cancer and adjacent normal tissues using integrative transcriptomics and proteomics identified chitinase‐3‐like protein 1 (CHI3L1) and MASP2 as biomarkers in HCC diagnosis. However, when diagnosed in combination, the detection for HCC was further enhanced (264). Analyzing patients with moderate and severe chronic hepatitis C, Tulio et al. identified five SNPs in regions critical for formation of MBL/MASP-2 complexes and C4 cleavage of MASP2 gene that resulted in high plasma levels of MASP-2 in hepatitis C patients (265). Mechanistic investigations into the determinants regulating MASP-2 expression *via in silico* analysis of the *MASP2* promoter regions revealed conservation of two putative Stat binding sites, StatA and StatB. *In vitro* analysis of hepatoma cell line HepG2 revealed double stranded StatB oligonucleotides, suggesting interaction of lectin and STAT signaling in liver diseases including fatty liver, fibrosis, and HCC (266). The diverse roles of MASP-2 documented in malignancies other than HCC warrant detailed further analysis into the roles of MASP-2 in adult and pediatric HCC.

### C4b-Binding Protein (C4BP)

C4BP is a fluid-phase regulatory component with potent inhibitory properties of the classical and lectin pathways of complement system (267) by providing cofactor activity for factor I-dependent degradation of C4b and C3b (268) (269) and accelerating decay of C3-convertases (269, 270). C4BP is synthesized primarily by hepatocytes (271) and activated monocytes (272). Synthesis of C4BP is enhanced in the presence of inflammatory cytokines such as IFN-γ, IL-1, IL-6, and TNF-α (273), with increased levels of C4BP in inflammatory diseases (274–277). Searching for biomarkers of colorectal cancer (CRC), especially the asymptomatic nascent tumors, Kopylov et al. (278) identified increased levels of C4BP as a potential biomarker in patients with CRC. In patients with non-metastatic CRC, C4BP levels correlated with several coagulation factors, suggesting risk factors for intravascular coagulation activation (279). Elevated levels of fully sialylated C4BP are also found in patients with epithelial ovarian cancer and can distinguish early cases of ovarian clear cell carcinoma from endometriomas (280). Profiling the pre-therapy serum proteome of patients with non-small cell lung cancer (NSCLC) to discover biomarkers and for patient-tailored therapeutics, Liu et al. applied shotgun and targeted proteomic analysis to identify relapse-related gene signatures. Results from the analysis identified a combination of C4BP, LRG1, and SAA or C4BP alone as determinants of disease prognosis, treatment optimization, and overtreatment prevention in patients with NSCLC (281). Indeed, NSCLC cells produce C4BP and provide significant protection from complement mediated tumor cell death (282). Another study also found increased serum C4BP levels in patients with NSCLC and showed strong associations with clinical staging (283). Thus, the ability of C4BP to regulate tumorigenesis in multiple organs and the liver as a primary source strongly suggests a role for C4BP in HCC. The proinflammatory cytokines IL-1, IL-6, and Oncostatin M all significantly upregulated C4BP expressions in the HepG-2 hepatoma cell line (284), suggesting an interplay between inflammation-driven regulation of complement components shielding the tumor from cytotoxic effectors. Tomes et al. further showed that C4BP not only binds strongly to necrotic cells but also limits DNA release from necrotic cells, inhibiting complement activation in both events. Persistence of necrotic core due to C4BP binding may have serious implications in cancer patients, manifesting with poor prognosis, enhanced tumorigenesis, progressive metastases, and emergence of chemoresistance (285). In keeping with the protective and tumor-augmenting roles of C4BP, Williams et al. showed that C4BP binds to CD154 and prevents CD40 mediated cholangiocyte apoptosis. Livers of patients with HCC showed enhanced expression and co-localization of C4BP and CD40, suggesting modulation of cholangiocyte survival in conditions of chronic inflammation and malignancy (286). Similarly, the hepatitis B virus X protein (HBx) affords protection of hepatoma cells from complement attack by upregulating C4BPα *via* activation of the transcription factor Sp1 (287). In tissues from patients with HCC, C4BPα expressions positively correlated with HBx, suggesting tumor-enhancing properties. Using protein-protein interaction networks and gene expression data from patient populations, Ardakani, et al. (288) identified C4BP as an important component of a common molecular relationship between HCC and liver cirrhosis. Identification of such networks and associated molecular connections are expected to serve as novel biomarkers and/or aid in the development of novel treatment strategies. The integral roles played by C4BP in regulating processes critical to tumor growth and progression make it an attractive target for developing interventional therapeutics.

### Complement Component C4 (C4)

C4 is the fourth component of the complement cascade, vital to several key roles in defense mechanisms, innate immune function, clearance of CICs, regulation of apoptotic bodies, and autoimmune processes (289, 290). Differentially altered levels of C4 are linked to inflammation in chronic liver diseases (291), metabolic syndrome (292), chronic urticaria (293), and autoimmune processes (294). Serum C4 levels can be used in early detection of HCC, particularly in HCV-infected patients with liver cirrhosis. Serum levels of complement C4 were detected at notably higher levels in the HCC group than in controls. Further analysis showed that a combination of AFP and C4 significantly improved the detection of HCC in HCV-related liver cirrhosis patients (295). More importantly, HCV proteins transcriptionally repress complement C4 expression in liver biopsy specimens from patients with HCV infection. mRNA levels of the two C4 isoforms C4a and C4b are also decreased in hepatocytes transfected with HCV RNA and in HCV core transgenic mice. Thus, the suppression of complement mediated immune responses promotes chronic HCV infection, fibrosis, and HCC (296). Investigating the impact of HBV infection on expression of serum C4 levels, Zhu et al. found that HBV similarly inhibits the expression of complement C3 and C4 *in vitro* and *in vivo* (297). Since AFP alone is used in clinical practice as a biomarker of HCC, Kim et al. performed global data mining using HCC proteomic databases to identify novel biomarkers. Alongside AFP, the data analysis revealed a set of other biomarkers including C4a (with ANLN and FLNB) that were proposed to further improve the screening of patients with HCC (298). Serum C4a/C4b also constitute clinically relevant candidate biomarkers in association with KNG1 and HPX, distinguishing patients with HCC and liver cirrhosis (299). Complement C4 also represents a component that can distinguish HCC and liver cirrhosis with the highest accuracy (300, 301). Increased levels of serum C4a were also found in HCV-infected alcoholic patients with progressive cirrhosis and HCC (302). As a precursor to development of HCC, patients with HBV or HCV infection are at greater risk, necessitating a specific biomarker with increased sensitivity. Dalal et al. identified increased C4a/C4b levels as a reliable marker in patients with HCV related end-stage liver disease (303). Thus, the direct participations in biological regulation of immune responses in HCC and the ability of differential expressions to distinguish patient populations as biomarkers signify complement C4/C4a/C4b as important targets for disease modulation and therapeutic targeting.

### Complement Factor H-Related Protein 1 (CFHL1)

Similar to CFH, CFHL1 is an immunoregulatory complement component produced primarily in the liver (304). CFHL1 is derived *via* alternative splicing of the N-terminal domain and shares negative regulatory functions of the alternative complement pathway similar to N terminus of CFH (305). Along with CFHL1, the complement factor H-related protein 1 (CFHR1) functions as a complement regulator by blocking C5 convertase activity and C5b surface deposition (306). CFHR1 also competes with CFHL1 for binding to C3b during CFH-regulation of immune processes (307). The role of CFHR in bladder cancer has been documented, showing the importance of the CFH family of proteins in oncogenesis (308, 309). In surgically resected tissues from HCC patients, decreased CFH mRNA expressions correlated with increased CpG site methylations (143). Furthermore, reduced CFHR3 expression was associated with tumorigenesis, cell proliferation, and activation of WNT and NOTCH signaling pathways (148). In this context, Feng et al. recently demonstrated that CFHL1 can be used as a potential prognostic biomarker in HCC. Analysis of tumor and peritumor specimens from patients with HCC showed downregulation of CFHL1 that was associated with worse time-to-recurrence of the cancer and reduced patient survival rates. This signifies the high prognostic value and potential biomarker capacity of CFHL1 in postoperative patients with HCC (310). The importance of CFHL1 in tumor biology, particularly in HCC, has recently been explored. Future studies will expectedly investigate the clinical efficacies of restoring CFHL1 levels to counter progressive oncogenesis.

### Complement Component 8A (C8A)

Complement component 8 alpha (C8A) is a late-phase component of the complement cascade and, along with C5, is involved in the formation of membrane attack complex (MAC). C8A is a liver-specific protein whose expression is regulated by hepatocyte nuclear factor 1α (HNF1α) (311). With relevance to HCC, C8A has been identified in the secretome of an HCC cell line, HEP3B. C8A was also identified as a putative biomarker in a study that investigated HCC–specific proteins enriched for cancer secretome followed by interactome analysis (242). Using genome-wide transcriptional profiling of patient specimens, 439 differentially expressed mRNAs (DEGs) and 214 long non-coding RNAs (lncRNAs; DELs) were identified in HCC. Multiple DELs correlated with tumor cell differentiation, thrombosis, AFP levels, and co-expressions of DEGs of complement cascade, including complement C8A (312). Similarly, Zhe L. et al. (313) utilized publicly available gene expression profiling datasets from the gene expression omnibus (GEO) to identify differentially expressed genes between tumor and adjacent healthy tissue, and found significant enrichment of genes involved in complement activation and coagulation cascade including C8a, C8b, and C6, in HCC specimens. Performing Ingenuity Pathway Analysis (IPA) of HCC gene expression data sets, Yin et al. identified uniquely decreased expression of C8A. Corresponding decreases in expression levels of other complement components including C1S, C2, C5, C6, C7, C8B, C8G, C9 were identified, strongly suggesting downregulation of key complement molecules during early stages of HCC (314). Mu Di et al. used ONCOMINE and TIMER to identify C6 as a candidate gene in diagnosis and prognosis that was associated with significantly decreased overall survival in patients with HCC (315). The regulatory roles of C8A participating in key functions of MAC formation and governing the fate of the tumor cell death can potentially be harnessed in understanding terminal complement processes and/or design of targeted therapeutics.

### CD46

CD46 is a membrane-bound complement regulatory protein (mCRP) expressed on the cell surfaces that restrains over-activation of the complement system and protects tissues from injury. CD46 primarily controls the alternative over classical pathway of complement activation. Besides its role as an mCRP, CD46 uniquely functions as a regulator of T cell mediated immune responses that may be relevant in the pathophysiology of HCC invasion and progression. Binding of CD46 on CD4^+^ T cells promotes differentiation to T regulatory phenotype (316) and dysregulated IL-10 production (317). While expression of CD46 on unconventional γδ T cells suppresses the production of IFN-γ and TNFα (318), CD4^+^ T cell ligation of CD46 results in production of IFN-γ (319). Thus, the duality of CD46 signaling in anti- and pro-tumoral functions necessitates a careful evaluation of its function in oncogenesis. In patients with ovarian and breast cancer, expression of CD46 is linked to shorter relapse periods and worse prognosis (320) (321); similar outcomes are observed in patients with CRC (322) and multiple myeloma (320). In patients with HBV-HCC, the HBx protein upregulates CD46 in hepatoma and human immortalized liver cells and affords protection from complement mediated cell lysis mechanisms (323). Investigations into CD46 distribution and expression patterns in HCC specimens showed a non-polarized membrane localization of CD46 in contrast to the basolateral expression in non-cancerous livers. This divergent expression pattern may allow HCC cells to escape complement-dependent cytotoxicity (324). In this regard, intratumoral and IV therapies that utilize the nonpathogenic oncolytic measles virus Emonston strain (MV-Edm) showed significant inhibition of tumor growth, survival benefits, and tumor regression in susceptible mice *via* CD46 targeting. This approach, therefore, represents a novel HCC gene therapy system (325). A similar approach using a fiber chimeric oncolytic adenovirus that targets CD46, SG635-p53, showed antitumor activity in Hep3B subcutaneous xenograft tumor models. Intratumoral injections of the adenovirus resulted in significant inhibition of tumor growth and survival of animals, suggesting a safe approach for HCC treatment (326). CD46 was also targeted using another oncolytic adenovirus, SG511, which was fused to the human RANTES/CCL5 gene and regulated by oxygen-dependent degradation domain (ODD). The chimeric SG511-CCL5-ODD showed significantly enhanced antitumor efficacy in HCC xenograft models in nude mice (327). The importance of the CD46 signaling pathway association with miRNA signatures in HCC was demonstrated *via* bioinformatic analysis. The authors performed complement-related gene expression profiling in tissue samples and found a total of 37 differentially regulated miRNA. Unsupervised hierarchical clustering analysis identified high CD46 expressions in HCC tissues, which negatively correlated with let-7b and miR-17 expression in HepG2 cells, suggesting important regulatory roles of CD46 in HCC *via* modulation of miRNA activities (328). Of note, upregulation of let-7 (329) and miR17 (330) has been associated with progressive carcinogenesis and poor prognosis of HCC. More importantly, the CD46 SNP rs2796267 was recently found to contribute to susceptibility and disease outcomes in HCC by modifying promoter activity. The rs2796267 AG/GG genotype was found to be associated with worse prognosis of resected patients with HCC (331). **Table 2** summarizes the various SNPs found in complement proteins relevant to the pathogenesis of HCC. To overcome the limitations associated with using monoclonal antibodies in cancer immunotherapies due to increased expressions of mCRPs, Geis et al. designed siRNAs for posttranscriptional gene knock down of CD46, CD55, and CD59 in tumor cell lines. The approach successfully reduced CD46 protein expression by 80% with a corresponding increase in CDC by 20%–30%, demonstrating sensitization of malignant cells to complement attack *via* siRNA mediated inhibition of mCRP as a means of cancer therapy (332). A concise summary of the complement proteins together with their biological functions and clinical implications is provided in **Table 3**.

**Table 2:** Summary of complement protein single nucleotide polymorphisms (SNPs) in hepatocellular carcinoma (HCC).

Complement protein	SNP	Biological function	Reference
**C2**	rs9267665	Increases the risk of HCC and Liver Cirrhosis (LC). Alters transcriptional activity of C2	Clifford et al. (192)
	rs2647073	Associated with HCC and LC	
	rs3997872	Associated with HCC but not with LC	
	rs10947223	Protects from HCC. Alters transcriptional activity of C2	Namgoong et al. (193)
	rs9279450	Protective effects against HCC and chronic hepatitis B (CHB)	
**CR1**	rs3811381	Increases risk of HBV-HCC and HBV-Chronic hepatitis B in males	Luo et al. (231)
	rs2274567	Increases risk of CHB, HBV-HCC in males	
**MBL2**	rs7096206	Influences the outcomes of HCC susceptibility, progressive tumor development, and clinical outcomes in HBV-HCC	Gu et al. (247)
	rs1800450	Modifies disease in patients after HBV infection, and affects the prognosis of patients with HBV-HCC	
	rs11003123	Risk factor for HCC development in the Chinese population	Wang et al. (196)
**MBL**	rs7096206	Associated with polymorphisms in VDR/VEGF and IL-18 which collectively confer susceptibility to HCC in the Asian population	Quan et al. (246)
	Codon 52, Codon 54, Codon 57	Associated with disease prognosis in patients with HBV, severe hepatitis B (SHB) or LC. Not associated with HCC prognosis	Xu et al. (250)
	Codon 54	Associated with symptomatic hepatitis B cirrhosis and in patients with spontaneous bacterial peritonitis (SBP)	Yuen et al. (251)
**MBL/MASP-2 complex**	p.D371Y	Involved in C4 cleavage of MASP2 gene and susceptibility to HCV infection. High levels of plasma MASP-2 are found in Hep-C patients	Tulio et al. (265)
**CD46**	rs2796267	Associated with susceptibility and disease outcomes in HCC by modifying promoter activity. Also defines grave prognosis of resected patients with HCC	Liu et al. (148)

**Table 3:** Summary of clinical and biological roles of complement proteins in hepatocellular carcinoma (HCC).

Complement proteins	Expression in HCC	Clinical implications in HCC	Biological functions	References
**CFH**	**Decreased**	Increases hepatocellular inflammation and injury Promotes enhanced tumorigenesis Poor overall and disease-free survival	Inactivates C3b and regulates activation of AP. Produced by epithelial endothelial cells	Weiler et al. (142) Laskowski et al. (119) Yang et al. (143)
**CFHR3**	**Decreased**	Increases cell proliferation and tumor burden Decreased cell apoptosis Poor overall and disease-free survival	Regulates WNT & NOTCH pathways. Prognostic predictor for targeted therapeutics in HCC	Liu et al. (147) Liu et al. (331) Pan et al. (149)
**C2**	**Decreased**	Worse prognosis time-to-recurrence of HCC Increases mortality and reduced survival times Promotes tumorigenesis and metastasis	Increases cytotoxicity of CD4^+^ T cells. Reduces M2 macrophage polarization. Regulates multiple signaling pathways.	Ning et al. (190) Fu et al. (191) Tian et al. (40)
**CR1**	**Decreased**	Increases hepatocellular inflammation and injury Increases grades of cirrhosis and HCC Contributes to decreased disease-free survival	Inhibits classical and AP pathways. Defective clearance of CICs. Potential therapeutic target in HCC	Chen et al. (183) Kanto et al. (229) Weisman et al. (234)
**MBL/MBL2**	**Decreased**	Enhances tumorigenesis and cancer burden Enhances PGE_2_ production and HCC progression Increases HSC activation and tumorigenesis Potential biomarker of diagnostic value	Promotes accumulation of MDSCs. Increases Treg function and activity Impairs CD8^+^ T cell cytotoxicity Enhances activation of HSCs	Rong et al. (241) Li et al. (243) Yoshino et al. (245) Gu et al. (247)
**CFHL1**	**Decreased**	Worse time-to-recurrence of HCC Increased cell proliferation and tumorigenesis Reduced overall and disease-free patient survival	Negatively regulates AP of C activation. Regulates C5b deposition & immunity. Correlates with CpG site methylations. Regulates WNT/NOTCH pathways	Zipfel & Skerka. (305) Heinen et al. (306) Yang et al. (143) Liu et al. (331)
**C8A**	**Decreased**	Worse time-to-recurrence of HCC. Increased cell proliferation and tumorigenesis. Reduced overall and disease-free patient survival	Promotes differentiation & thrombosis. Decreased levels of C8A correlate with early HCC	Yao et al. (312) Yin et al. (313) Mu et al. (315)
**C4**	**Decreased**	Contributes to augmented liver inflammation Diagnostic marker for HCV related HCC. Biomarker for HCV infection, fibrosis and HCC	Distinguishes HCC & cirrhosis with highest accuracy Low C4 levels promote fibrosis & HCC	Potter et al. (291) Ali et al. (295) Banerjee et al. (296)
**CD59**	**Increased**	Decreases complement-mediated cell lysis Decreases apoptosis and increased tumor burden Worse overall and disease-free survival	Increased resistance of cancer cells Regulates Smad7 formation and Smad2/3 phosphorylation. Modulates TGF-β signaling	Fishelson et al. (151) Watson et al. (156) Abdel-Latif et al. (161) Lan & Wu (166)
**CD46**	**Increased**	Increases tumor growth & decreases regression Shorter relapse periods and worse prognosis Decreases overall and disease-free survival	Decreases complement cytotoxicity. Promotes differentiation of Tregs. Modulates HCC via miRNA activities	Sherbenou et al. (320) Kinugasa et al. (324) Lu et al. (328)
**C1q**	**Increased**	Increases cancer cell migration and proliferation Increases tumorigenesis and tumor burden. Poor overall and disease-free survival	Contributes to tumor angiogenesis Promotes cancer cell metastasis Enhances invasiveness of cancer cells	Hong et al. (169) Bulla et al. (186) Hoffken et al. (180) Ho et al. (189)
**C3**	**Increased**	Increases chemoresistance to therapeutics Promotes angiogenesis and metastasis Potential biomarker of diagnosis & prognosis	Promotes immunosuppressive. TME Suppresses anti-tumor CD8^+^ T cells. Increases M2 macrophages & MDSCs	Pio et al. (195) Wang et al. (249) Leung et al. (203)
**C5**	**Increased**	Increases metastasis and EMT of cancer cells Modulates proliferative & apoptosis pathways Enhances dissemination of cancer tumor cells	Promotes immunosuppressive TME. Decreases CD8^+^ T cell cytotoxicity. Enhances functions of MDSCs	Dai et al. (210) Medler et al. (213) Kusmartsev et al. (218)
**C4BP**	**Increased**	Promotes progressive metastases & tumor burden Positively correlates with HCC and liver cirrhosis Poor prognosis and HCC chemoresistance	Persistently maintains necrotic core. Modulates cholangiocyte survival. Shields tumors from cytotoxic cells	Phillips et al. (284) Tomes et al. (285) Williams et al. (286)
**MASP-2**	**Variable**	Diagnostic marker for HCC No clear role defined for MASP-2	Potentially regulates fibrosis and HCC	Ding et al. (264) Unterberger et al. (266)

## Complement Proteins as Regulators of Liver Metastases

The aforementioned components and receptors of the complement cascade not only regulate hepatic neoplasia but promote early events of metastases involving increased tumor cell motility, invasiveness, and intravasation. The extra- and intra-hepatic metastatic spread remains one of the major hurdles in improving health related quality of life and long-term survival in patients with metastatic HCC and therefore is one of the most prevalent form of cancers with poor prognosis. HCC cells that survive immune-mediated clearance continue to proliferate and reserve the capacity to generate secondary tumors. Within this framework, perturbation of the complement cascade facilitates dissemination of the tumor cells *via* triggering intracellular EMT pathways and transition to a highly motile cellular phenotype. Recent studies have correlated the C5a/C5AR1 axis with increased angiogenesis and metastasis promoting factors that induce EMT (333) and liver metastasis (334). In HCC, C5AR1 increases cell invasiveness by enhancing Snail and decreasing E-cadherin and Claudin-1 expressions (209). The ability of C5AR1 to facilitate metastasis was also linked to suppression of CD8^+^ and CD4^+^ T-cell responses *via* recruitment of immature myeloid cells and generation of Tregs. This study also showed that pharmacologic blockade or genetic ablation of C5AR1 prevented metastatic potential of cancer cells (220). Expression of C5AR1 on TAMs conferred M2 polarization in colon cancer and enhanced liver metastatic lesions affirming a central role for C5AR1 in metastatic spread; importantly, genetic loss of C5ar1 severely impaired the metastatic ability of colon cancer cells (211). Genetic ablation of other complement proteins such as C3 was also shown to have profound inhibitory effects on primary tumor growth and metastasis correlating to increased numbers of IFNγ^+^/TNFα^+^/IL10^+^ CD4^+^ and CD8^+^ T-cells (335). The ability of complement C3 to function in conjunction with EMT contributing towards metastasis is shown by the ability of TWIST1 to regulate C3 expression in tumor cells (209). Cumulatively, the complement components work in synchrony as a “dark knight–a watchful protector” offering immune surveillance and regulating tumorigenesis and metastatic potential of the transformed oncogenic cells.

Juxtaposing these components are the membrane-bound and soluble complement regulatory factors that protect tumor cells from immune mediated cytotoxicity. Incidentally, high expressions of CD46, CD55, and CD59 are homogenously expressed and positively correlate with increased metastatic tumor cells in the liver of patients with colorectal (336) and other cancers with poor prognosis (321, 337). Inhibitory factors such as CFH were also shown to be highly expressed in exosomes of the metastatic cells (EV-CFH) resulting in increased migratory and invasive capacity of liver cancer cells. Blocking EV-CFH with a tumor specific anti-CFH antibody showed reduction in liver tumor promoting potentials and a potential therapeutic target (338). Directly “complementing” these pro-oncogenic functions, the complement cascade also interacts with the coagulation system resulting in a hyper-coagulable state and survival of tumor cells. In this context, C5a stimulates neutrophils to release tissue factor (339) while C3a induces platelet aggregation and activation (340), both processes culminating in a prothrombotic environment. Furthermore, the ability of neutrophil derived C3AR1 to form neutrophil extracellular trap (NET) drives tumorigenesis (341) and potentially enhances metastatic capacities. It is therefore important to consider these interactive mutually synergistic pathways in the design of novel therapeutics targeting HCC.

## Therapeutic Targeting of Complement System: A Reality?

**Figure 3:**
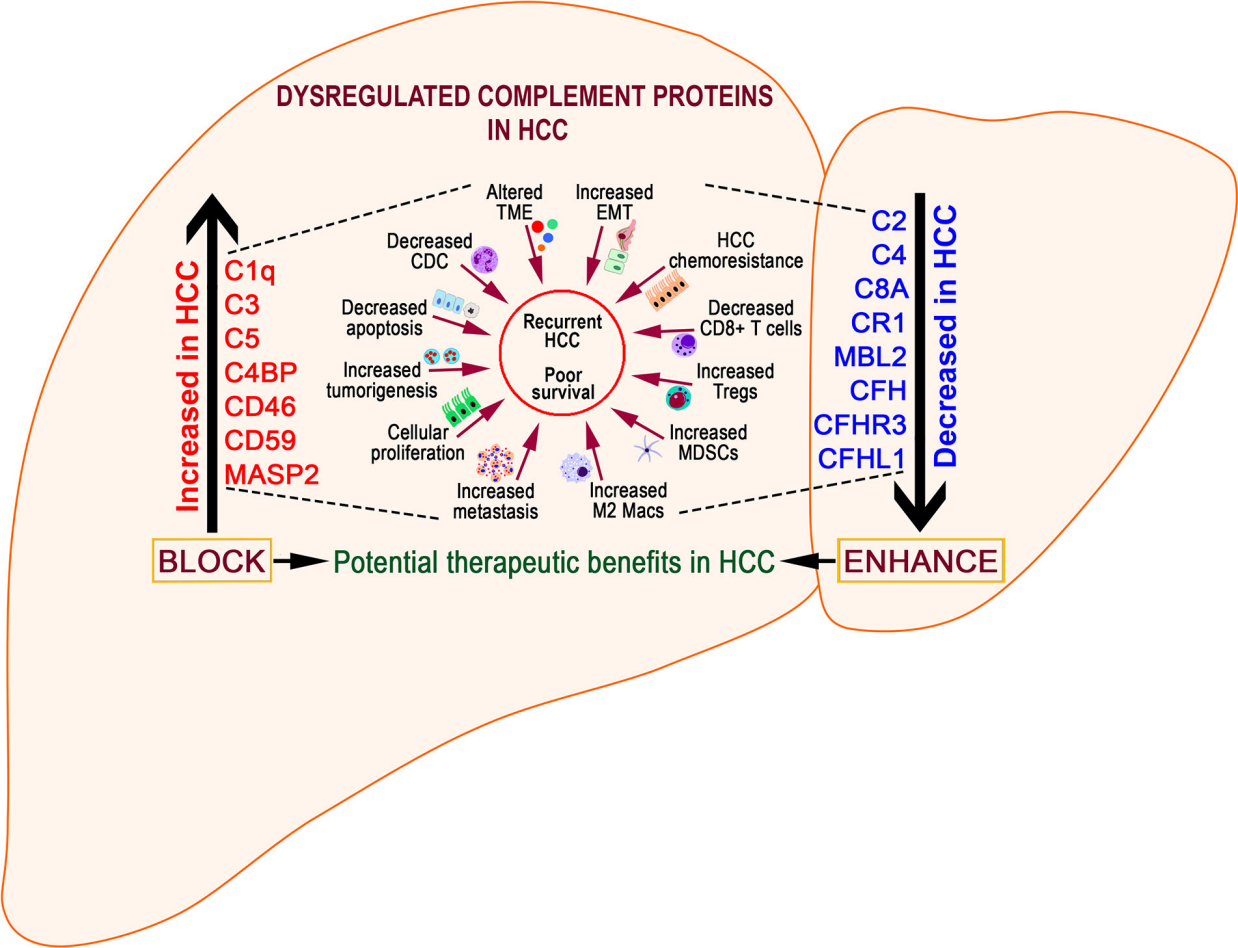
Dysregulated expressions of complement components orchestrate the pathobiology of hepatocellular carcinoma. Breakdown of the tightly controlled activation and regulatory component signals of the complement system results in dysregulation of the normal homeostatic cellular processes within the liver microenvironment. Increased levels of C1q, C3, C5, C4BP, CD46, CD59, and MASP2 and decreased levels of C2, C4, C8A, CR1, MBL2, CFH, CFHR3, and CFHL1 with potential immunoregulatory functions contribute to increased cell proliferation, metastasis, EMT, altered immune cell functions, etc. resulting in recurrent episodes and/or poor overall or disease-free survival in patients with HCC. Images from Motifolio drawing toolkit software (http://motifolio.com) were used for rendering the figure.

The myriad effects of complement molecules in regulating the TME and molecular and cellular effectors of immunopathogenic mechanisms driving HCC may offer new avenues to develop complement-based therapeutics. **Figure 3** depicts the influence of differential complement protein expressions in regulating key pathobiological functions promoting oncogenesis in HCC and provides a platform for therapeutic interventions. In particular, the immune-based therapies have raised concerns and skepticism over failures to produce clinically meaningful disease-modulating effects in cancers. Some anticancer immunotherapies that inhibit PD-1 and/or PD-L1, such as Nivolumab and Pembrolizumab, are currently used to treat unresectable HCC. They can also induce complement activation due to their affinities for C1q and Fc receptors (342, 343); increased C1q levels have been shown to augment liver damage. In this context, C1-INH, approved by the FDA for treatment of hereditary angioedema, has been shown to block the classical activation pathway *via* C1q inhibition (344). Blocking C1q activity may thus represent a beneficial approach in regulating tumorigenesis in HCC while preserving the functions of other complement pathways. In addition to C1q, the roles of other downstream complement molecules such as C5a have been extensively studied in HCC. Inhibition of C5a within the TME without deleterious effects on additional complement dependent defenses has previously been proposed (344). In HCC, C5a activation was shown to induce EMT *via* inhibition of claudin-1 and activation of ERK1/2 pathway. Therefore, targeting C5a generation *via* anti-C5 antibodies (Eculizumab) or blocking C5a-C5aR interaction using a receptor antagonist (PMX-53) that are currently in clinical trials for acute myocardial infarction or rheumatoid arthritis respectively, may serve as promising therapeutic candidates for HCC. Other components of the complement cascade such as C3, C3a/C3b could also be targeted using inhibitors such as the Compostatin/POT-4, currently in clinical trials to treat age related macular degeneration (123). These studies highlight the importance of therapeutic targeting of complement as a novel therapeutic strategy for HCC. In parallel, complement dependent cytotoxicity (CDC) and antibody dependent cellular cytotoxicity (ADCC) are the leading cause of cell death when treating tumors/cancer cells with monoclonal antibodies. Several factors and/or etiopathogenic agents have been associated with tumor progression in HCC. Circulating apoptosis inhibitor of macrophage (AIM) is one such element that was recently described by Maehara et al. to play a role in activation of the complement cascade on the cell surface of tumorigenic, not normal, hepatocytes due to defective endocytosis (345). The authors showed that membrane bound AIM accumulation resulted in C3 activation *in vivo* and was detrimental for viability of cancer cells in HCC *via* CDC cascade. The fundamental dogma in complement biology is a skewing towards enhanced inflammation, with therapeutic approaches designed primarily towards inactivating the complement cascade; however, in situations of tumorigenesis, local stimulation of complement may be advantageous. In this regard the recently described anti-CD20 mAb currently in development: HuMax CD20, HuMax CD38, and HuMaX ZP3, have been demonstrated to increase CDC potency. Anti-CD20 mAb such as Rituximab may therefore prove beneficial in patients with HCC (346–348).

As discussed in the previous section, several factors contribute to the etiopathogenesis of HCC. One such factor, the oncolytic viruses, have been associated with tumorigenesis in HCC and are known to activate complement cascade. Recently, Kim et al., utilized Pexastimogene devacirepvec (Pexa-vec), an oncolytic virus, and showed its ability to induce complement-mediated cancer cell cytotoxicity in rabbits, resulting in improved survival in tumor bearing animals (349). Survival benefits were also achieved in patients with advanced hepatocellular carcinoma that were treated with Pexa-Vec (350). Due to significant success and promising clinical activity of the oncolytic virus, Pexa-vec has recently been tested along with Nivolumab to target tumor cells in HCC (348, 351). In conjunction with our review on the factors of complement cascade and the role of activated components in promoting HCC oncogenesis, we provide a platform identifying various molecules of the complement pathway as potential therapeutic targets in treating patients with HCC and fostering improved survival.

## Concluding Remarks and the Impact of COVID-19

HCC continues to be a grave prognostic feature for patients with advanced liver disease of varying etiologies. While early diagnosis remains the mainstay of appropriate medical and surgical approaches, the mostly uncharacteristic features of HCC circumvent early disease diagnosis. The worldwide prevalence, lack of available therapeutic modalities, and rapid progression to severely compromised liver functions urgently necessitate identification and interrogation of newer mechanisms towards better treatment approaches. This urgent need is also dictated by the only available treatment of HCC that relies on liver transplantation, which inherently suffers from shortage of donor livers, higher costs, risk of tumor recurrence, etc.

The demand and need for non-surgical systemic therapies to effectively manage and treat HCC are greater than ever due to the evolving COVID-19 pandemic that has greatly overwhelmed the healthcare system. HCC patients are especially vulnerable due to the decreased allocation of healthcare resources including limited access to operating rooms, deferrals and delays in curative surgery and ablation therapies. The ever-changing scenario of the pandemic, disparity amongst nations in infection rates and limited data of COVID-19 infected HCC patients dictates ongoing efforts in liver oncology. HCC patients require repeated hospital visits, experience social and nosocomial contacts, risks posed by the prevalence of asymptomatic COVID-19 carriers in the community, treatment-related immunosuppression and more importantly treatment delays (352). A recent study reported 21.5% of patients with HCC experienced a significant treatment delay: longer than 1 month in 2020 compared to 2019 (353) as well as a significant drop in number of follow up patients visits (354). Moreover, the significant burden on healthcare providers and resource-intensive protocols have offered little guidance in addressing treatment strategies (352). Therefore, care providers must ensure appropriate surveillance, treatment, and monitoring of patients with HCC and continue to provide therapeutic avenues as in non-COVID-19 pandemic. A system to triage HCC patients where resources are limited should be adapted along with efforts to eliminate the virus in patients with confirmed COVID-19 infection (355). Amongst the many etiopathogenic factors known to cause or promote HCC, the real impact of COVID-19 pandemic or the SARS-CoV-2 virus itself in HCC patients remains unknown. Recent reports have indicated about 15%–54% of patients infected with the virus have hepatic injury and elevated levels of transaminases (356). It is therefore plausible that HCC patients infected with COVID-19 may experience exacerbated disease symptoms and predisposed to increased risk of secondary infections leading to significant morbidities or early mortality. Indeed, the risk factors that predict higher overall mortality in patients with chronic liver disease and COVID-19 are alcohol-related liver disease, decompensated cirrhosis and HCC (357). Using retrospective cohorts, many studies have associated increased biomarkers of liver injury (ALT, AST, GGT) to SARS-CoV-2 infection (358–360) with worsened disease responses in HCC and other cancers. Although the fundamental and intrinsic regulators remain unknown, increased injury responses have been ascribed to direct cytopathic effects of the SARS-CoV-2 virus on hepatocytes and/or cholangiocytes, hypoxia, immune-mediated hepatitis, etc. (356, 361). Standard care treatments such as antivirals and antibiotics prescribed to treat COVID-19 infection have also been linked with increased risk for hepatotoxicity and elevated liver enzymes. To overcome these new challenges and design effective treatment strategies, combination therapies that utilize existing or newly designed immunomodulators targeting complement cascade proteins described herein with immune checkpoint inhibitors may hold significant promise and provide novel therapeutic strategies to treat HCC patients with superimposed COVID-19 infection.

HCC is an immunogenic cancer characterized by chronic inflammation, fibrosis, and cirrhosis. Dysregulated immune responses constitute a major risk factor for HCC. The chronic inflammation, secondary to persistent liver damage, promotes immune cell activation and increased apoptosis. These events enhance tumorigenesis *via* cell stress, epigenetic modifications, altered mitochondrial metabolism, and activation of cellular senescence pathways. Combined, these biological phenomena directly regulate the high density of liver resident macrophages, NK cells, innate lymphocytes, etc. constituting the immune system as a potential target for managing and treating HCC. Central to these processes, recent studies have assigned multifaceted roles for complement molecules in the immunoregulation of HCC-TME. In this review, we have aimed to decipher the mechanistic roles of complement system in immune dysregulation and oncogenesis in HCC. Our review also describes several components of the complement cascade as potential targets for development of therapeutics. This is highly relevant in the context of limited chemotherapeutic measures that include Sorafenib, Lenvatinib and Regorafenib which currently form the standard care for advanced non-resectable HCC.

**Figure 4:**
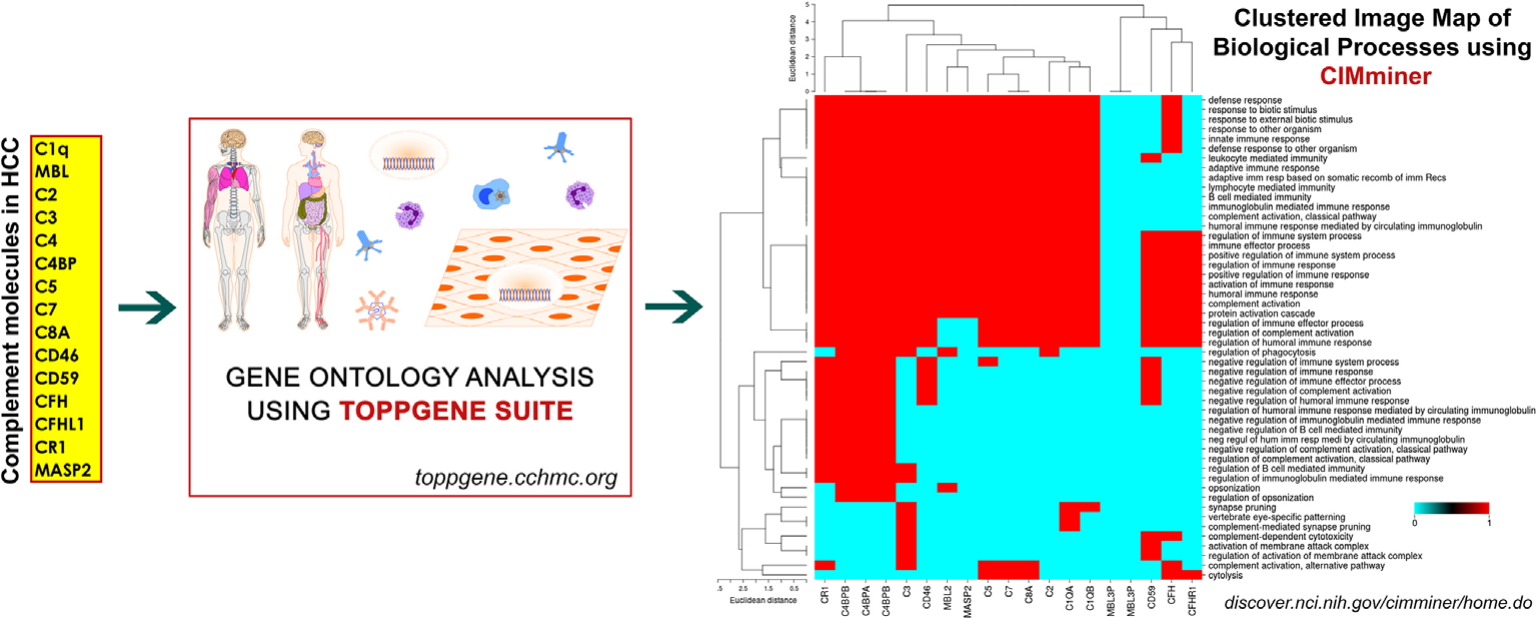
Bioinformatics analysis identifies dominant signatures of immune–mediated biological processes. Supervised gene ontology (GO) annotation analysis of complement components in hepatocellular carcinoma (HCC) was performed *via* ToppGene Suite portal (http://toppgene.cchmc.org) for *in-silico* enrichment of biological processes with a threshold False Discovery Rate (FDR) corrected *P* value of <0.05. ToppGene Suite is a freely available online tool used for functional enrichment, prioritization of candidate genes using transcriptome, ontology, phenotype, proteome, and functional annotations. GO: biological processes identified by ToppGene were further subjected to functional enrichment using CIMminer (https://discover.nci.nih.gov/cimminer/home.do). Red areas depicted in the heatmap show closely related biological processes linked to immunity that are shared by a major group of complement molecules shown on the horizontal axis.

**Figure 5:**
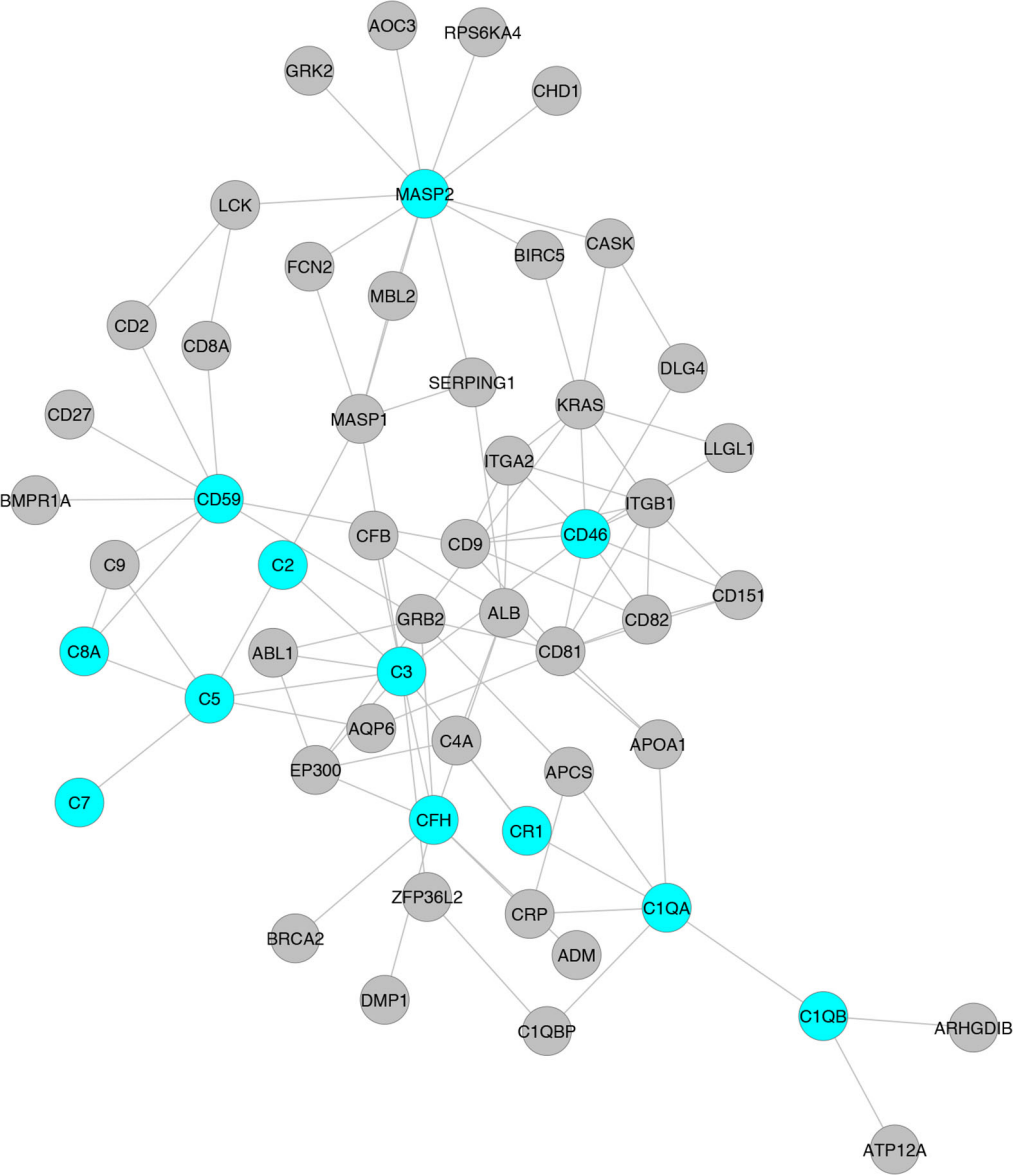
Protein-protein interaction network analysis identifies biological relatedness of complement components to regulators of hepatocellular carcinoma (HCC) pathogenesis. Complement molecules of relevance to HCC were subjected to protein-protein interaction (PPI) network analysis using the network-based gene prioritization algorithm, ToppGenet of the ToppGene Suite (http://toppgene.cchmc.org). ToppGenet identifies and prioritizes candidate genes based on functional annotations, similar expressions, and network and topographical features. A Step Size of 6 and the Prioritization method of k-Step Markov were used as default analytical parameters. The Cytoscape-compatible ToppGenet output file was used to generate the graphical network. The first shell of 41 interacting proteins (grey color) associated directly with the input complement proteins (blue) in the PPI were generated by Cytoscape.

We have made an effort to elucidate how complement system regulates the activation of cellular and molecular responses including NK cells, DCs, MDSCs, TAMs, TANs, cytokines, and chemokines that form the first line of defense. Impaired complement activation alters the anti-tumorigenic immunological responses involved in halting the progression and expansion of the tumor, leading to HCC. Efforts to treat HCC *via* modulation of the pro-tumorigenic immune response in TME have been explored, but failure of these therapies in producing clinically meaningful effects limits their use. Elevated expressions of various complement components including C1q, C8a, and anaphylatoxins have been demonstrated in HCC tumors. Targeting these components either by specific inhibitors or antagonists in synergism with existing therapies has a great potential to treat HCC. In contrast to promoting tumor progression, components such as C2 and CFH exhibit tumor-suppressive effects and better prognosis in HCC patients. Furthermore, complement C3, C3a, C4, C4a, and C7 have been identified as biomarkers in HCC diagnosis while C7 and CFH are recognized for their critical roles in mediating stemness of tumor-initiating cells. Similarly, complement C5 regulates EMT, cell migration, and invasion, MBL activates stellate cells, CR1 clears complement CICs, C4BP acts as a complement inhibitor, and CD59 suppresses C3 activation and MAC formation while loss of CFHL1 corresponds to poor time-to-recurrence and overall survival rates. Given these divergent roles, future efforts should be directed toward developing strategies that selectively target or inhibit the tumorigenic effects of complement components while promoting or retaining their anti-tumorigenic effects. Development of novel and targeted therapeutics also benefits from an array of integrated bioinformatics analysis using either a biologically validated known set of genes or a discovery module that employs either large-scale transcriptomic or proteomic techniques. As an initial approach to identify molecular partners and biological processes linked to Complement, we subjected complement components found in HCC (C1q, C2, C3, C4BP, C5, C7, C8A, CD46, etc.) to bioinformatics analysis. Analyzing the genes using ToppGene database (http://toppgene.cchmc.org) with a threshold of *P*<0.05, we identified several biological processes that were further clustered using CIMminer (https://discover.nci.nih.gov/cimminer/home.do). A significant number of complement molecules defined several dominant pathways linked to activation and regulation of innate and adaptive immune systems emphasizing the importance of complement-driven immunopathogenesis (**Figure 4**). Protein-protein interaction network (**Figure 5**) generated using ToppGenet (https://toppgene.cchmc.org) and complement genes showed close associations with several molecules that either positively (ADM, APOA1, ATP12A, BIRC5, CFB, CHD1, DLG4, GRB2, ITGA2, ITGB1, KRAS, LCK) or negatively (APCS, BRCA2, CASK, CD27, CD81, CD82, DMP1, FCN2, GRK2, LLGL1) regulate the pathogenesis of HCC. Regulation of these diverse processes by complement molecules may further propel identification of novel interventional targets for drug development and therapeutic interventions. Thus, the complement cascade serves as a link between the innate and adaptive immune system, activating immune cells critical to drive HCC pathogenesis. With the current understandings of complement molecules as oncogenic drivers, targeted therapies could be developed independently or in combination with existing first and second line of HCC therapies. In summary, a deeper understanding of the mechanistic role of tumor complement components in these pro- and anti-tumorigenic pathways supplemented by advanced bioinformatics approaches are expected to foster the design and development of effective clinical treatments for HCC.

## Author Contributions

AM and UT equally contributed towards design and manuscript drafting. SA, RM, SN, and AB compiled the literature and assisted in drafting the manuscript. RM analyzed complement genes using bioinformatics tools. PS conceived the original idea and completed the final version of the manuscript. All authors contributed to the article and approved the submitted version.

## Funding

This work was supported in part by NIH P30 DK078392 of the Digestive Diseases Research Core Center in Cincinnati.

## Conflict of Interest

The authors declare that the research was conducted in the absence of any commercial or financial relationships that could be construed as a potential conflict of interest.
